# Endophytic Fungi: A Treasure Trove of Antifungal Metabolites

**DOI:** 10.3390/microorganisms12091903

**Published:** 2024-09-18

**Authors:** Sanjai Saxena, Laurent Dufossé, Sunil K. Deshmukh, Hemraj Chhipa, Manish Kumar Gupta

**Affiliations:** 1 Department of Biotechnology, Thapar Institute of Engineering and Technology, Patiala 147004, Punjab, India; ssaxena@thapar.edu; 2 Agpharm Bioinnovations LLP, Incubatee: Science and Technology Entrepreneurs Park (STEP), Thapar Institute of Engineering and Technology, Patiala 147004, Punjab, India; 3 Chimie et Biotechnologie des Produits Naturels (ChemBioPro Lab) & ESIROI Agroalimentaire, Université de la Réunion, 15 Avenue René Cassin, CS 92003, F-97744 Saint-Denis, France; 4 R&D Division, Greenvention Biotech Pvt. Ltd., Uruli Kanchan 412202, Maharashtra, India; 5 College of Horticulture and Forestry, Agriculture University Kota, Jhalawar 322360, Rajasthan, India; hrchhipa8@gmail.com; 6 SGT College of Pharmacy, SGT University, Gurugram 122505, Haryana, India; mkgupta5@gmail.com

**Keywords:** natural products, resistance, metabolites, drug discovery, volatiles, chemicals

## Abstract

Emerging and reemerging fungal infections are very common in nosocomial and non-nosocomial settings in people having poor immunogenic profiles either due to hematopoietic stem cell transplants or are using immunomodulators to treat chronic inflammatory disease or autoimmune disorders, undergoing cancer therapy or suffering from an immune weakening disease like HIV. The refractory behavior of opportunistic fungi has necessitated the discovery of unconventional antifungals. The emergence of black fungus infection during COVID-19 also triggered the antifungal discovery program. Natural products are one of the alternative sources of antifungals. Endophytic fungi reside and co-evolve within their host plants and, therefore, offer a unique bioresource of novel chemical scaffolds with an array of bioactivities. Hence, immense possibilities exist that these unique chemical scaffolds expressed by the endophytic fungi may play a crucial role in overcoming the burgeoning antimicrobial resistance. These chemical scaffolds so expressed by these endophytic fungi comprise an array of chemical classes beginning from cyclic peptides, sesquiterpenoids, phenols, anthraquinones, coumarins, etc. In this study, endophytic fungi reported in the last six years (2018–2023) have been explored to document the antifungal entities they produce. Approximately 244 antifungal metabolites have been documented in this period by different groups of fungi existing as endophytes. Various aspects of these antifungal metabolites, such as antifungal potential and their chemical structures, have been presented. Yet another unique aspect of this review is the exploration of volatile antifungal compounds produced by these endophytic fungi. Further strategies like epigenetic modifications by chemical as well as biological methods and OSMAC to induce the silent gene clusters have also been presented to generate unprecedented bioactive compounds from these endophytic fungi.

## 1. Introduction

Fungi catapulted into the spotlight as fountainheads of bioactive substances following the discovery of Penicillin produced from *Penicillium notatum*. Since that pivotal moment, fungi have become an integral entity in the landscape of pharmaceutical research and development, igniting the creation of diverse drug classes, including antibiotics, immunomodulators, antifungals, and anti-thrombotic agents [1].

On a parallel path, endophytic fungi, stealthily residing within plant tissues sans any overt signs of their presence, have emerged as key players over the past two decades. Their significance lies in producing a spectrum of bioactive compounds and signal molecules due to their co-existence and evolution with their host plants. This unique relationship endows them with the inherent capability to generate both the presumed phytochemicals of their hosts and their own congeners.

Thus, endophytic fungi have become crucial contributors to fungal biodiversity, continually unraveling extraordinary bioactive properties across diverse global landscapes. Simultaneously, there has been a surge in the resistance exhibited by microorganisms, including pathogenic fungi, against antimicrobials, which is attributed to the excessive and inappropriate use of antibiotics. This phenomenon has given rise to superbugs, posing a huge threat within clinical settings. Consequently, there is an escalating demand for untried antibacterial and antifungal agents to combat drug-resistant microorganisms. Previously, fungal infections were underestimated, but they are now garnering serious attention due to their potential to cause untreatable secondary infections in hospitalized individuals and patients dealing with conditions like cancer and immunomodulatory disorders. As a result, this review centers’ on endophytic fungi sourced from medicinal plants in the past six years (2018–2023), focusing on those demonstrating promising antifungal activity or antifungal agents with the potential to advance into the drug development pipeline. Generally, in a natural, product-based antimicrobial discovery program, a variety of panels of test microorganisms (plant/humans/animals) are tested to understand the potential of the new chemical entity for pharmaceutical or agrochemical development. The mode of action of the bioactive secondary metabolites produced by fungi generally remains the same irrespective of the type of pathogen (plant or animal). Gliotoxin was first isolated from the wood fungus *Gliocladium fimbriatum*. It exhibits a remarkable anti-fungal activity, which is mediated via ROS (Reactive oxygen species) production, creating a redox disbalance, resulting in oxidative stress and leading to the death of fungi [2]. Despite being too toxic to enter into the drug development pipelines, several similar structures have found their way into drug development. Dehydrogliotoxin is a natural analog of gliotoxin with reduced toxicity, but the biological activity is retained. It has shown promise for the development of an antifungal agent with reduced cytotoxicity apart from potential in cancer therapy [3]. Another derivative of gliotoxin comprises Bis(methylthio)gliotoxin, which also possesses antifungal activity [4]. Griseofulvin, produced by *Penicillium griseofulvum*, stands out as a unique example that serves a dual role in agriculture as well as pharmaceuticals. The mechanism for both actions is the same; via disruption of cell division by targeting the microtubules, which are crucial for the cell division of fungi as well as other mammalian cells [5,6]. Several classes of bioactive compounds have been produced by endophytic fungi, which hold the potential to be used as antifungals for phytopathogenic as well as animal/human pathogenic fungi. However, due to climacteric changes in the environment, the line between the phytopathogenic fungi and opportunistic fungi causing human infections is receding. There have been several reports in the recent past wherein human infections have been caused by opportunistic phytopathogens. Thus, in this review paper, we shall be classifying the potential compounds obtained from endophytic fungi of medicinal plants based on different chemical classes and their mode of antifungal action.

## 2. Coumarins

Coumarins constitute a group of compounds that are characterized by a 2H-chromen-2-one core structure. The 2H-chromen-2-one core structure is formed by fusing a benzene ring with a pyrone ring. Diverse forms of coumarins are found in nature due to variations in their structures arising from substitutions on the coumarin nucleus. Coumarins and isocoumarins are specialized derivatives of polyketides sharing a common biosynthetic origin and pathway [7].

Coumarins exert their antifungal effect due to several mechanisms involving cell membrane damage, enzyme inhibition leading to cell wall synthesis inhibition, disruption of electron transport chain causing mitochondrial dysfunction, pro-oxidant action by generating and accumulation of ROS and disruption of signal transduction pathways, which are responsible for growth, differentiation, and pathogenicity of fungi. The coumarins produced by fungal endophytes represent a diverse and potent class of antifungal agents [8].

**Figure 1:**
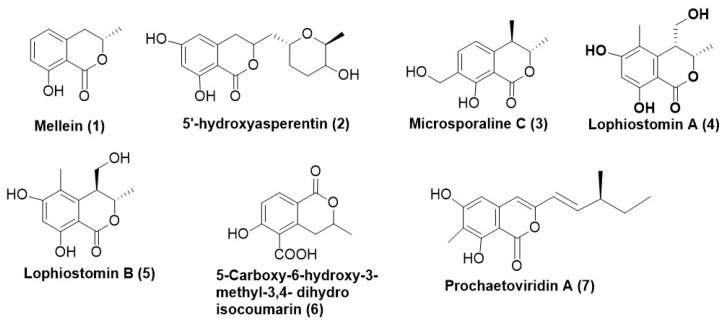
Antifungal coumarins reported from endophytic fungi (**1**–**7**).

Mellein (**1**) (Figure 1) was purified from *Aspergillus* sp. SPH2 recovered from the stem of *Bethencourt iapalmensis*, an endemic plant in Abona, Spain. Compound (**1**) displayed antifungal activity with EC_50_ values of 340, 440, and 290 µg/mL, respectively, against *F. oxysporum*, *A. alternata* and *B. cinerea* in mycelial growth inhibition assay [9]. 5′-hydroxyasperentin (**2**) (Figure 1) is an isocoumarin derivative isolated from *Cladosporium cladosporioides*, existing as an endophyte in the plant *Zygophyllum mandavillei* exhibiting antifungal activity against *Aspergillus flavus* and *Fusarium solani* with MIC of 15.62 μg/mL and 7.81 μg/mL [10].

One unprecedented isocoumarin microsporaline C (**3**) (Figure 1) was isolated from the endophytic fungus *Pestalotiopsis microspora* SC3082. Compound (**3**) displayed an average antifungal activity with a MIC value of 12.5 μg/mL against *C. albicans* ATCC 10321 [11]. Dihydrocoumarin congeners Lophiostomin A (**4**) and Lophiostomin B (**5**) (Figure 1) were isolated from the endophytic fungus *Lophiostoma* sp. Signf 10 obtained from *Siratia grosvenorii*. Both Lophiostomin A (**4**) and B (**5**) expressed moderate inhibitory activity towards fungi and inhibited spore germination of *Magnaporthe oryzae* [12]. 5-carboxy-6-hydroxy-3-methyl-3,4-dihydroisocoumarin (**6**) (Figure 1) was isolated from *Xylaria* sp. existing as an endophyte in *Casearia sylvestris* growing at the Experimental Ecological Station, Mogi Guaçu, São Paulo, Brazil. TLC bioautography revealed that the compound (**7**) exhibited potent antifungal activity against *C. cladosporioides *and *C. sphaerospermum* [13].

Prochaetoviridin A (**7**) (Figure 1), an isocoumarin and an indole alkaloid derivative isolated from *Chaetomium globosum* CDW7, existing as an endophyte in *Ginkgo biloba* exhibited moderate antifungal activity against a fungal test panel comprising of *Sclerotinia sclerotiorum*, *Fusarium graminearum*, *Phytophthora capsici*, *F. moniliforme*, and *Botrytis cinerea*. The inhibition of test panel cultures ranged between 13.7% to 39.0% at 20 µg/mL [14].

## 3. Cytochalasins (Aka Cytochalasans)

Cytochalasins constitute a group of complex secondary metabolites produced by fungal endophytes. Their basic structure comprises a 16-membered macrocyclic ring fused to an isoindole moiety. The variations in their structures arise from different side chains and substituents attached to this core, which influence their specific biological activities, which range from anti-tumor, anti-microbial, cytotoxic, and HIV protease inhibition [15]. The general mechanism of action of cytochalasins is based on their ability to disrupt the actin cytoskeleton of eukaryotic cells, thereby inhibiting actin polymerization and disrupting the dynamic nature of the cytoskeleton [16]. The downstream effects of this disruption affect cellular processes, such as inhibition of cell division, impact on membrane functions, and effects on motility and cell shape. They also inhibit the secretion of the enzyme produced by the fungi to grow and proliferate, thereby inducing antifungal action. This has been proven experimentally in the case of Cytochalasin A using the *Aspergillus nidulans* model [17].

**Figure 2:**
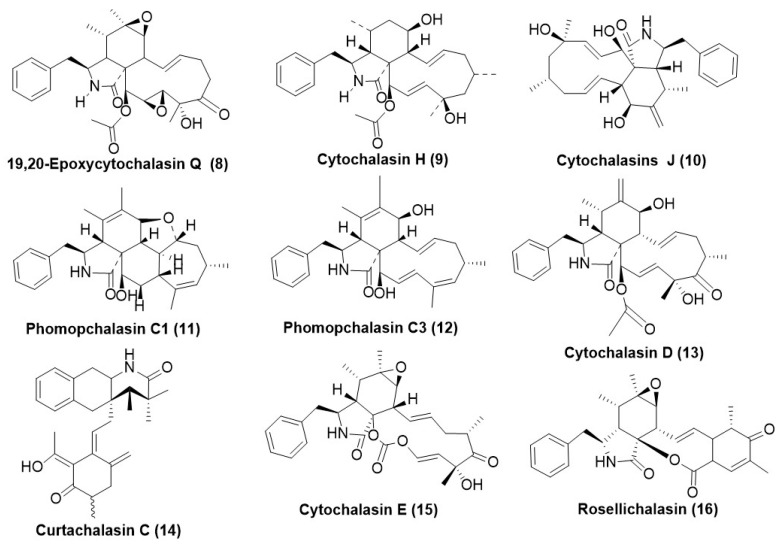
Structures and Metabolites of Cytochalasin class produced by fungal endophytes (**8**–**16**).

Recently, 19,20-epoxy cytochalasin Q (ECQ) (**8**) (Figure 2) was isolated from the endophytic fungus *Xylaria* sp. BCC1067 was found to disrupt the actin filament dynamics of the model yeast *Saccharomyces cerevisiae* [18]. It has also been identified as an inducer and potential substrate of key multidrug efflux pump substrates *S. cerevisiae* ScPdr5 and *Candida albicans* Cacdr 1 [19,20]. This has opened avenues for developing new antifungal armamentariums, as they could potentially overcome the antifungal drug resistance in multidrug-resistant fungi exhibiting efflux transporters. Hence, these could possibly be used as a synergist of the current drugs to overcome the phenomenon of resistance towards antifungals.

Fungal genera comprising *Ascochyta*, *Aspergillus*, *Helminothosporium*, *Phoma*, *Tubercularia*, *Xylaria*, and *Diaporthe* have been reported to produce diverse cytochalasins. Cytochalasin H (**9**) and J (**10**) (Figure 2) were isolated from endophytic *Diaporthe miriciae*, residing in plants *Copaifora pubifora* and *Melocactus ernestii* collected from the Amazon Forest. Cytochalasin H (**9**) at a concentration of 148.08 µg/mL exhibited a 73% reduction in the growth of *P. obscurans* after 144 hrs. However, Cyrochalasin J (**10**) exerted only a 36% reduction in the growth of *P. obscurans* after 144 hrs. Hormesis (minor stimulation of mycelial growth) was also exhibited by Compounds (**9**) and (**10**) in certain fungal species such as *Colletotrichum acutatum*, *Colletotrichum fragariae*, *Colletotrichum gloeosporioides*, *Botrytis cinerea*, and *Fusarium oxysporum*. *Phomopsis viticola* also exhibited inhibition by cytochalasin’s H (**9**) and J (**10**), exhibiting a growth reduction of 63% and 58%, respectively [21].

Phomochalasin C1 (**11**) and C3 (**12**) (Figure 2) have been obtained from *Phomopsis* sp. xz-18, an endophyte residing in *Camptotheca acuminata* stems existing in Jiangshi Natural Reserve, China. Compound (**11**) exhibited average antifungal activity against *Aspergillus niger* ACCC3005 with inhibition zone diameters of 8 mm at 50 μg/disc (5 mm), and compound phomochalasin C3 (**12**) displayed obvious antifungal activity against *Candida albicans* AS2.538, giving a 10 mm zone of clearance by disk diffusion assay. Amphotericin B was used as a positive control with a 15 mm zone of inhibition in both *Candida albicans* AS2.538 and *Aspergillus niger* ACCC3005 [22].

Cytochalasin D (**13**) (Figure 2) was purified from *Xylaria* species existing as an endophyte in *Paullinia cupana*. Namely, *Xylaria* sp. 214, from Brazil, had fungistatic activity against *C. gloeosporioides* at a concentration of 100 µg/mL. It exhibited a reduction in mycelial growth by 38.8%, while the MIC was 2.46 µM/mL. Positive controls captan and difenoconazole exhibited a MIC of 16.63 and 0.02 µM/mL, respectively [23]. Another endophytic *Xylaria* cf. *curta* expressed Curtachalasin C (**14**) (Figure 2), which has a unique bridged 6/6/6/6 ring system in its structure. It possesses antifungal resistance reversal properties as it inhibited fluconazole-resistant *Candida albicans* by 50% when mixed in a ratio of 8:5 and exhibited a much significant improvement as compared to the use of fluconazole alone. Individual use of Curtachalasin C (**14**) at a concentration of 128 µg/mL did not exhibit any inhibition of fluconazole-resistant *Candida* sp., suggesting its possible non-antifungal action [24]. *Xylaria* sp. BCC 1067, an endophytic fungus, produced the compound 19,20-epoxy cytochalasin Q (ECQ) (**8**) (Figure 2). ECQ (**8**) exhibited a MIC_50_ of 410 and 55 µg/mL against the model yeast *Saccharomyces cerevisiae* wildtype and ScΔpdr5 strains, respectively. ECQ (**8**) was identified as an inducer and a substrate for multidrug efflux pumps and acts via targeting actin filaments, thereby disrupting the actin dynamics of yeast cells. An additional mechanism by which it exerts its antifungal action is its pro-oxidant behavior, resulting in the accumulation of high ROS, eventually damaging the plasma membrane, and resulting in decreased yeast cell survival. Compound (**9**) also synergized with azoles to revert resistance against *S. cerevisiae* expressing multidrug transporters. Thus, this compound can be a potential solution to combat antifungal drug resistance [25]. Another endophytic fungus belonging to *Diaporthe* sp. GDG-118 was isolated from the plant *Sophora tonkinensis*, which was recovered from Heichi City, China, and produced cytochalasin E (**15**) (Figure 2). Cytochalasin E (**15**) exhibited potent antifungal activity against *Alternaria oleracea*, *Pestalotiopsis theae*, and *Colletotrichum capsica*, with MIC values of 3.125, 1.56 and 1.56 µg/mL, respectively [26]. Rosellichalasin (**16**) (Figure 2) has been isolated from an endophytic fungus, *Aspergillus capensis* CanS-34A, associated with the plant *Brassica napus* collected from Wuhan City, Hubei, China. The compound rosellichalasin (**16**) inhibited the test panel fungi (*B. cinerea*, *M. fructicola*, *S. sclerotiorum*, and *S. trifoliorum*) with EC_50_ values ranging from 2.46 to 40.31 μg/mL while the positive control, fungicide Procymidone exhibited EC_50_ values in the range of 79 to 2.78 μg/mL [27].

## 4. Polyketides

Polyketides constitute diverse classes of secondary metabolites, which are found in plants, microbial systems (bacteria and fungi), and animals. These are generally synthesized by a battery of enzymes known as Polyketide synthases (PKS), which generally condense acyl-CoA units successively to generate diverse and complex chemical scaffolds with unique biological activities [28]. Polyketides exert antifungal action broadly by (a) disrupting the fungal cell membrane integrity, (b) inhibiting the cell wall synthesis, (c) interfering with the transcription and translation processes, and (d) disrupting the mitochondrial function. Amphotericin B is already a proven polyene macrolide polyketide, which has a potential antifungal activity via binding to the ergosterol moiety, thereby disrupting the cell membrane structure. Amphotericin B is a broad-spectrum antifungal that treats invasive fungal infections caused by *Candida*, *Aspergillus*, and *Cryptococcus* species [29]. On the other hand, inhibition of the biosynthesis of fungal cell walls could also be responsible for its growth inhibition. This mechanism basically relies on the inhibition of enzyme 1,3-β-d-glucan synthase, which synthesizes glucan, an integral component of the fungal cell wall. Echinocandins generally work by inhibiting the formation of glucan by the enzyme 1,3-β-d-glucan synthase [30]. Caspofungin is a non-ribosomal polyketide derivative, which selectively targets the 1,3-β-d-glucan synthase and obstructs the fungal cell wall biosynthesis. Another example of polyketide which interferes with mitochondrial function is strobilurins. Strobilurins bind to the cytochrome bc1 complex, thereby blocking the electron transport chain and ATP synthesis, rendering the cell depleted in energy and ultimately leading to cell death [31]. An array of endophytic fungi from ascomycetes have been reported to produce polyketides as a single culture or co-culture [32].

**Figure 3:**
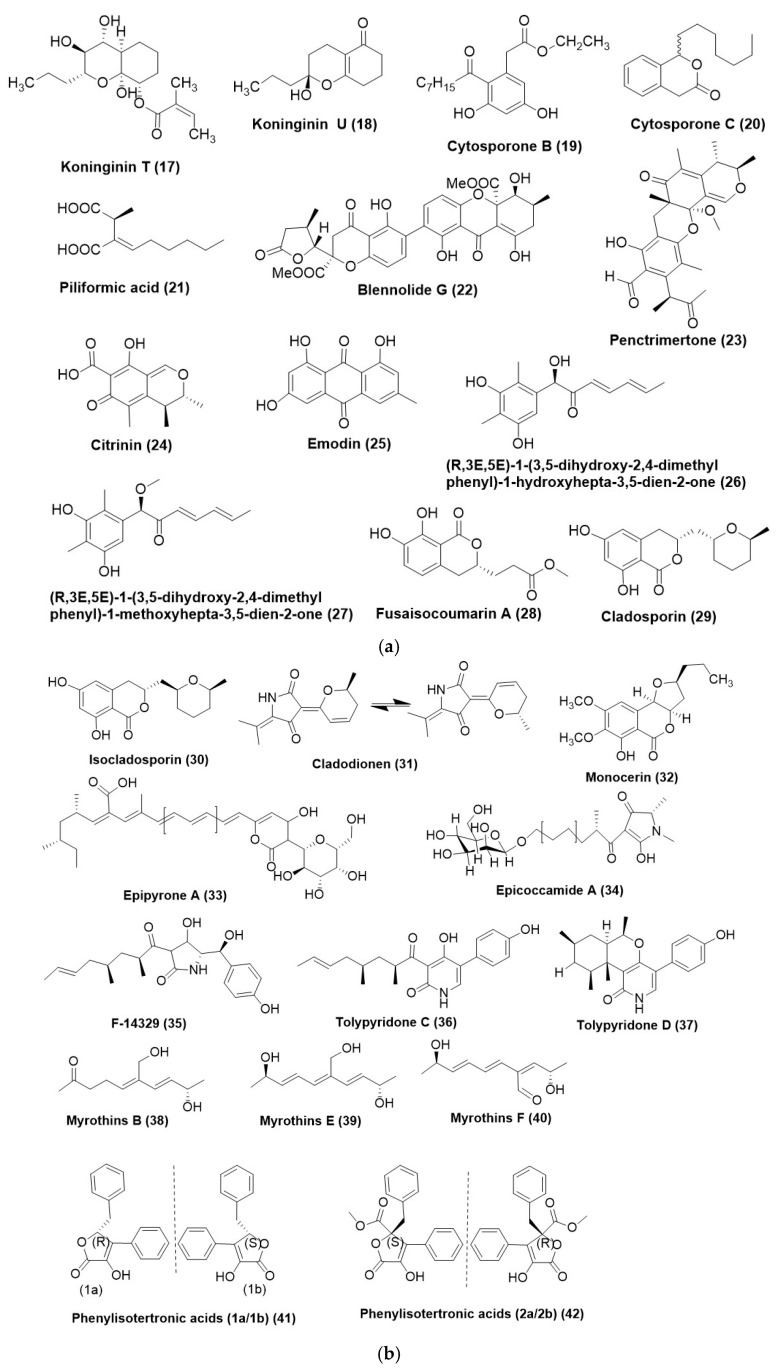

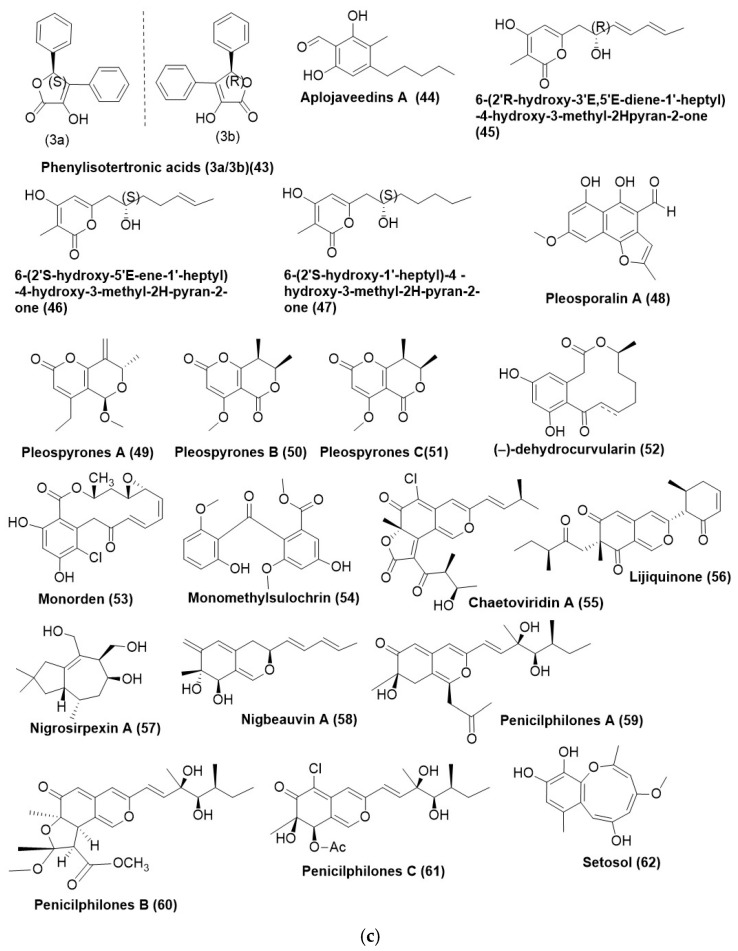
(**a**) Antifungal polyketides produced by fungal endophytes (**17**–**29**); (**b**) Antifungal polyketides produced by fungal endophytes (**30**–**42**); (**c**) Antifungal polyketides produced by fungal endophytes (**43**–**62**).

*Phomopsis stipata*, an endophyte of the plant *Styrax camporum* inhabiting Sao Paulo, Brazil, produced two unreported polyketides named koninginin T (**17**) and U (**18**) (Figure 3a). Compound (**17**) showed moderate antifungal activity against *Cladosporium cladosporioides*, but compound (**18**) was cent percent fungicidal to *C. sphaerospermum* and *C. cladosporioides* at a MIC of 100 μg/mL as compared to 25 μg/mL Nystatin used as a positive control [33]. Cytosporone B (**19**) (Figure 3a) is a fungal polyketide that was purified from *Phomopsis phyllanthicola* A658 was isolated from the tissues of *Pogostemon cablin*, widely cultivated in Guangdong province in China. MIC exhibited by Cytosporone B (**19**) was 105 µg/mL as compared to the positive control prochloraz (95 µg/mL) activity against *Geotrichum citri-aurantii*. Cytosporone B (**19**) was also found to regulate the genetic expression of *G. citri-aurantii*, as it upregulated 1412 unigenes and 2128 downregulated unigenes. In the in vivo studies, cytosporone B (**19**) exhibited a protective action on sugar oranges inoculated with *Geotrichum citri-aurantii* at the concentration of 500 µg/mL, which was comparable to prochloraz at the concentration of 250 µg/mL [34]. Cytosporone C (**20**) (Figure 3a) has been isolated from *Diaporthe eucaluptorum* KY-9, which exists as an endophyte in *Melia azedarach* in Yangling, Shaanxi Province, China, when grown on solid rice culture. It exhibits antifungal activity [35].

*Xylaria* sp. 249, existing as an endophyte in *Paullinia cupana* in Brazil, was found to produce Piliformic acid (**21**) (Figure 3a), which exhibited fungistatic activity against *C. gloeosporioides* at a concentration of 100 µg/mL. The reduction in the mycelial growth was 51.3%, while the MIC was 0.62 µg/mL against *C. gloeosporioides.* Positive controls captan and difenoconazole exhibited a MIC of 4.99 µg/mL and 0.0008 µg/mL respectively [23].

Blennolide G (**22**) (Figure 3a) has been isolated from an endophytic fungus, *Talaromyces stipitatus* DgCr2 2.1b recovered from the roots of *Duguetia stelechantha growing* in the campus of Universidade Federal do Amazonas, Manaus City, Amazonas State, Brazil. Blennolide G (**22**) exhibited MIC of 125 µg/mL against *Candida tropicalis* and *Candida albicans* when compared to the positive control fluconazole which had a MIC of 5 µg/mL [36].

Penctrimertone (**23**) (Figure 3a), a novel citrinin dimer bearing a 6/6/6/6 tetracyclic ring scaffold, was purified from *Penicillium* sp. T2-11, existing as an endophyte in the rhizomes of *Gastrodia elata*, was collected from Zhaotong, Yunnan Province, China. Compound (**23**) exhibited moderate antifungal activity with MIC of 4 µg/mL while against *C. albicans* while positive control nystatin with MIC of 2 µg/mL [37].

Two polyketides, citrinin (**24**) and emodin (**25**) (Figure 3a), were isolated from *Penicillium citrinum* DBR-9, an endophytic fungus residing inside the root tubers of the plant *Stephania kwangsiensis* from Guangxi Institute of Botany, Guilin, Guangxi, China. Citrinin (**24**) displayed antifungal activity against plant pathogenic fungus viz. *Alternaria citri*, *Colletotrichum capsica*, *Ceratocystis paradoxa*, *Cochliobolus miyabeanus*, *Diaporthe citri*, *Exserohilum turcicum*, and *Pestalotiopsis theae* with IC_50_ values in the range of 3.1 to 123.1 µg/mL and exhibited highest activity toward the pathogenic fungi *Alternaria citri*. Emodin (**25**) showed antifungal activity against six pathogenic fungi viz. *Bipolaris maydis*, *C. paradoxa*, *C. miyabeanus*, *D. citri*, *E. turcicum*, and *Phytophthora parasitica* var. *nicotianae* with IC_50_ values in the range of 3.0 to 141.0 μg/mL while it exhibited the highest activity against the pathogenic fungi *B. maydis*. Emodin (**25**) affected the colony morphology by destroying the cell membrane integrity and influencing the protein synthesis in the cells of the *Phytophthora parasitica* var. *nicotianae* [38].

Two structurally unprecedented polyketides (R,3E,5E)-1-(3,5-dihydroxy-2,4-dimethyl phenyl)-1-hydroxy hepta-3,5-dien-2-one (**26**), (R,3E,5E)-1-(3,5-dihydroxy-2,4-dimethylphenyl)-1-methoxy hepta-3,5-dien-2-one (**27**) (Figure 3a), were purified from *Trichoderma afroharzianum* and endophytic fungus associated with *Ficus elastica*. The site of the collection of *F. elastica* was Liaocheng University Arboretum, Liaocheng, Shandong Province of China. Compound (**26**) exhibited potent antifungal activity towards *B. cinerea*, *F. oxysporum*, *f.* sp. *nicotianae*, and *C. legendarium*, with MIC values of 8, 8, and 32 μg/mL, respectively. Compound (**27**) also displayed antifungal activity with MIC values of 16, 16, and 32 μg/mL, respectively, toward *B. cinerea*, *F. oxysporum*, f. sp. *nicotianae*, and *C. lagenarium* [39].

One polyketide metabolite, fusaisocoumarin A (**28**) (Figure 3a), was isolated from the endophytic fungus *Fusarium verticillioides* strain WF18e living as an endophyte in fresh leaves of *Mentha piperita* L., family Labiatae (Lamiaceae) collected from Izmir, Turkey. The antifungal activity of the compound (**28**) against a test panel of fungi comprising *Aspergillus austroafricanus* strain EIS1, *Aspergillus versicolor* strain EIS2, *Phoma fungicola* strain AT100, *Aspergillus tubingensis* strain AN103, and *C. albicans* ATCC 90029 exhibited a MIC in range of 0.3–0.4 μg/mL. The positive control, Amphotericin B, in a MIC range of 0.5–0.8 μg/mL [40]. Cladosporin (**29**) (Figure 3a) is a polyketide isolated from *Cladosporium cladosporioides*, an endophytic fungus isolated from *Zygophyllum mandavillei*. The MIC of Cladosporin (**29**) against *Aspergillus flavus* and *Fusarium solani* was found to be 500 μg/mL and 62.5 μg/mL, respectively, as compared to Amphotericin B exhibiting a MIC of 0.49 μg/mL. Isocladosporin (**30**) (Figure 3b) was a diastereoisomer of cladosporin at C-14 and also exhibits antifungal activity against *A. flavus* and *F. solani*, with a MIC of 15.62 μg/mL and 7.81 μg/mL, respectively [10]. Cladodionen (**31**) (Figure 3b) was isolated from *Cladosporium sphaerospermum* WBS017, an endophytic fungus which was isolated from bulbs of *Fritillaria unibracteata* var. *wabuensis*. The antifungal activity of the compound (**31**) against *Ustilgo maydis* was assessed by agar plate diffusion assay using a concentration of 100 μg/plate while the positive controls tested comprised of Hygromycin B (50 μg/plate) and Nystatin (100 μg/plate). It was observed that Compound (**31**) exhibited a stronger antifungal activity by giving an inhibition zone diameter of 0.97 ± 0.05 cm as against Hygromycin B, which exhibited an inhibition zone diameter of 0.83 ± 0.05 cm. However, the zone of inhibition, compound (**31**), was weaker compared to nystatin, which exhibited an inhibition zone of 3.27 ± 0.05 cm [41].

Monocerin (**32**) (Figure 3b) has been isolated from a root endophytic fungus, *Drechslera* sp. strain 678. It exhibited a moderate inhibitory effect against *S. sclerotiorum* and *B. cinerea* within two days of exposure when used at 100 µg/mL [42].

Epipyrone A (**33**) and Epicoccamide A (**34**) (Figure 3b) were isolated from *Epicoccum nigrum* MK214079, an endophytic fungus residing in the leaves of *Salix* sp. from the Caucasus mountains, Lago-Naki, Russia. The MIC value of compound (**33**) and compound (**34**) against *Ustaligo maydis* AB33 with MIC values of 1.004 μg/mL and 1.003 μg/mL, respectively, while the positive control was nystatin exhibited a MIC of 0.018 μg/mL [43]. Compounds F-14329 (**35**), tolypyridone C (**36**), and tolypyridone D (**37**) (Figure 3b) were purified from *Tolypocladium* sp. 49Y was isolated from the leaf of *Acorus tatar-inowii*, and the plant material was collected from Baiyun Mountain, Guangzhou, Guangdong Province, China. Compounds (**36**) and (**37**) showed potent antifungal activity against *Cryptococcus neoformans* with a MIC of 6.25 μM (2.22 μg/mL). On the other hand, compound (**35**) displayed poor antifungal activity towards *C. neoformans* with a MIC at 8.35 μg/mL [44].

Some compounds named myrothins B (**38**), E (**39**), and F (**40**) (Figure 3b) produced by *Myrothecium* sp. BS-31, an endophyte isolated from *Panax notoginseng* from an experimental plantation in Anning, Yunnan. Compounds (**38**) and (**39**) showed weak activity against *Fusarium oxysporum* and *Phoma herbarum* with a MIC of 128 μg/mL. Compound (**40**) displayed weak activity to *F. oxysporum* with a MIC of 128 μg/mL. The positive control nystatin showed antifungal activity with MICs at 28–56 μg/mL [45].

Four hitherto unknown phenylisotertronic acids (1a/1b) (**41**), (2a/2b) (**42**) (Figure 3b), and (3a/3b) (**43**) (Figure 3c) were obtained from *Phyllosticta* sp. J13-2-12Y, an endophyte associated with the leaves of *Acorus tatar-inowii*, collected from Guangxi Medicinal Botanical Garden, Guangxi Province, China, along with two known ones (2b and 3b) (**42**) and (**43**). Compounds 1a/1b (**41**), 2a/2b (**42**), and 3a/3b (**43**) were found active against *C. albicans* with MIC values of 2.2, 32, 4, 64, and 128 µg/mL respectively (positive control itraconazole MIC 0.5 µg/mL) [46].

A new polyketide aplojaveediins A (**44**) (Figure 3c) was characterized by *Aplosporella javeedii*, an endophytic fungus isolated from the plant *Orychophragmus violaceus*, collected around Beijing, China. Compound (**44**) displayed antifungal activity against the hyphae form of *C. albicans* strain ATCC 24433 with an inhibition diameter of 8 mm at a concentration of 1 mM. It was also found to be active against the yeast *Saccharomyces cerevisiae* with an inhibition diameter of 18 mm. The MIC of compound (**44**) against the hyphae form of *C. albicans* strain ATCC 24433 in a liquid medium was 100 µM as determined by the micro broth dilution assay. Incubation of cells of the hyphae form of *C. albicans* strain ATCC 24433 with compound (**44**) at 400 µM resulted in a rapid decrease of viability by 3.5-log over a period of 6 h, after which a plateau was reached. In contrast, the positive control hygromycin B (474 µM = 4-fold MIC), which has antifungal activity against *C. candida*, exhibited only a largely static growth inhibitory effect [47].

Three polyketides exhibiting substituted pyran-2-one core, namely, -(2′R-hydroxy-3′E,5′E-diene-1′-heptyl)-4-hydroxy-3-methyl-2Hpyran-2-one (**45**), 6-(2′S-hydroxy-5′E-ene-1′-heptyl)-4-hydroxy-3-methyl-2H-pyran-2-one (**46**), and 6-(2′S-hydroxy-1′-heptyl)-4 -hydroxy-3-methyl-2H-pyran-2-one (**47**) (Figure 3c) were isolated from root endophytic fungus *Penicillium ochrochloronthe* existing in *Taxus media* on Qingfeng Mountain, Chongqing, China. The antifungal activity test panel comprised *Fusarium graminearum*, *Cercospora arachidicola*, *Alternaria solani*, *Cylindrocladium parasiticum*, *Bipolaris carbonum*, *Alternaria alternata* f. sp. *Mali*, *Cercospora personata*, *Botrytis cinerea*, *Ustilago scitaminea*, *Rhizoctonia cerealis*, *Helminthosporium maydis*, *Colletotrichum graminicola*, *Exserohilum turcicum*, *Alternaria alternata*, *Colletotrichum gloeosporioides*, *Sclerotinia sclerotiorum*, *Colletotrichum orbiculare*, *Botrytis fabiopsis*, *Alternaria brassicae*, and *Ascochyta gossypii.* Compound (**45**) exhibited a MIC in the range of 12.5 to 100 μg/mL, while the compounds (**46**) and (**47**) displayed a MIC range of 12.5 to >100 μg/mL. Ketoconazole, the positive control, exhibited a MIC in the range of 0.78–6.25 μg/mL [48].

A heptaketide, pleosporalin A (**48**) (Figure 3c), was isolated from *Pleosporales* sp. F46 exists as an endophyte in the *Mahonia fortune* collected from Qingdao, China. As compared to the positive control Fluconazole (MIC_80_: 2.0 µg/mL) compound (**48**) exhibited a poor antifungal potential with a MIC_80_ of 128 µg/mL [49]. *Pleosporales* sp. Sigrf05, an endophyte of *Siraitia grosvenorii* inhabiting Guangxi Province of China, produced unfamiliar chlamydosporol derivatives named pleospyrones A-C (**49**–**51**) (Figure 3c). Germination of spore of *Magnaporthe oryzae* by compounds (**49**–**51**) exhibited an IC_50_ value of 98.73, 47.77, and 51.08 μg/mL respectively [50].

(−)-dehydrocurvularin (**52**) (Figure 3c) was reported from endophytic *Penicillium sumatrense* GZWMJZ-313 and exhibited antifungal activity against *C. albicans* ATCC10231 and *C. glabrata* ATCC2001 with MIC of >128 μg/mL and 8 μg/mL respectively while the positive control ketoconazole was 4 and 2 μg/mL respectively [51].

The antifungal compound activity of monorden (**53**) (Figure 3c), an inhibitor of heat shock protein 90 (Hsp90), was purified from *Humicola pulvericola* JS-0112. Isolated from the root of *Ixeris repens* collected from Silmi-do, Incheon. Monorden (**53**) inhibited the mycelial growth of oomycetous fungus *P. infestans*, *P. cactorum*, *P. cambivora*, *P. capsica*, *P. cinnamomic*, *Pythium ultimum*, *P. helicolis*, and *P. graminicola* exhibiting a MIC range of 0.078–2.5 µg/mL. Excluding *Phytophthora infestans*, all the oomycetes present in the test panel exhibited MIC values < 0.39 µg/mL. It also displayed good antifungal activity against tree pathogens *Cryphonectria parasitica*, *Taphrina wiesneri*, and *Valsa kunzei* with MIC values of 1.56 µg/mL. Monorden (**53**) effectively suppressed the development of rice blast caused by *Magnaporthe oryzae*, rice sheath blight caused by *R. solani*, and wheat leaf rust caused by *Puccinia recondita* in a dose-dependent manner in virulence assays. Monorden (**53**) exhibited a dose-dependent and effective control of rice blast disease. At a concentration of 500, 250, and 125 µg/mL, the control of the rice blast was 90%, 78.8%, and 25%, respectively. Monorden (**53**) effectively controls the cucumber damping off disease caused by *P. ultimum*. It exhibited a higher control efficacy of Monorden (IC_50_: 18.44 µg/mL) than the commercial fungicide Chlorothalonil (IC_50_: 40.1 µg/mL) [52].

Monomethylsulchrin (**54**) (Figure 3c) was purified from *Rhizopus* sp., an endophyte associated with plant *Astragalus membranaceus*. The compound (**54**) exhibited potential antifungal activity against *Fusarium moniliforme*, *Alternaria brassicae* and *P. anthracnose* in a MIC range of 32–128 µg/mL. Nystatin used as the positive control exhibited MIC in the 16–64 µg/mL [53].

Azaphilones comprise a diverse group of naturally occurring polyketide metabolites. Their distinctive feature is the presence of pyranoquinone, or pyrone-quinone core structure conjugated with various side chains, thus giving rise to many derivatives. The core structure of Azaphilones basically comprises a bicyclic system incorporating a six-membered pyrone ring fused with a five-membered lactone or lactam ring. They have a variety of substituents, including methyl, hydroxyl, and alkyl chains. They possess multiple stereocenters, and their complexity significantly affects their biological activity [54].

*Penicillium sclerotiorum*, an endophyte isolated from the mangrove plants, has been reported to produce sclerotinia, an azaphilone with significant antifungal activity, which inhibits the growth of *Botrytis cinerea*, a common fungal pathogen and hence suggests potential application as an agricultural fungicide [55]. The antifungal mechanism of action of Azaphilones is generally attributed to their ability to interfere with the integrity and function of fungal cell membranes and also the inhibition of enzymes, which are essential for the growth and proliferation of fungal cells [56]. Azaphilones have a propensity to form adducts with amino acids and protein, and this process is known as Michael’s addition [57]. The covalent bond formation with the biomolecules renders them inactive and disrupts the cellular function of the cells, eventually leading to cell death.

Chaetoviridin A (**55**) (Figure 3c), an azaphilone isolated from an endophyte of *Ginkgo biloba*, *Chaetomium globosum* CDW7, exhibited potential antifungal activity. At a concentration of 20 µg/mL, it inhibited a test panel comprising *Sclerotinia sclerotiorum*, *Fusarium graminearum*, *Phytophthora capsici*, *F. moniliforme*, and *Botrytis cinerea* in the range of 97.8% to 59.2%. *S. sclerotiorum* was specifically tested against the compound (**55**) with carbendazim as a positive control with a concentration of 0.17 µg/mL, where it exhibited an EC_50_ value of 1.97 µg/mL. However, during in vivo testing, on an infected rape plant, compound (**55**) exhibited a similar efficiency of 45.2% and 69.2% at an application rate of 100 and 200 µg/mL, respectively, as against 44.6 and 69.2% protection efficiency of positive control carbendazim at the same application rate [14].

*Ascomycete* sp. F53, existing as an endophyte in *Taxus yunnanensis* (Chinese yew), was subjected to genome mining. It revealed the presence of 35 putative biosynthetic gene clusters, which exhibited a close homology to the azaphilone biosynthesis pathway. This led to the identification of a novel entity, lijiquinone (**56**) (Figure 3c), exhibiting an antifungal activity against *Cryptococcus neoformans* and *Candida albicans* with IC_50_ of 53.9 µg/mL and 30.21 µg/mL respectively [58]. Unprecedented pulvilloric acid-type azaphilone Nigrosirpexin A (**57**) (Figure 3c) was isolated from *Nigrospora oryzae* co-cultured with *Irpex lacteus*. The MIC of nigrosirpexin A (**57**) was 6.2 μg/mL for *Irpex lacteus* compared to the positive control Cycloheximide exhibiting a MIC of 8.6 μg/mL while it showed a MIC of 64 µg/mL for *N. oryzae* [59]. The co-culture of *Nigrospora oryzae* and *Beauveria bassiana*, the endophytes of the seeds of *Dendrobium officinale*, yielded azaphilone nigbeauvin A (**58**) (Figure 3c). Compound (**58**) displayed antifungal activity against the co-cultured fungus *B. bassiana* and *N. oryzae*, with MICs of 128 and 512 μg/mL, respectively [60].

Highly oxygenated Azaphilones, Penicilphilones A-C (**59**–**61**) (Figure 3c), have been reported from the endophytic fungus *Penicillium* sp. LZUC-S1. These have exhibited antifungal activity against *Rhizoctonia solani*, *Fusarium oxysporum*, *Penicillium citrinum*, and *P. melonis*. Carbendazin was used as a positive control in the antifungal assay. Penicilphilones A (**59**) and C (**61**) exhibited a potent antifungal activity. Penicilphilone A (**59**) exhibited antifungal activity between 49.8% and 62.4% at a concentration of 50 µg/mL. Penicilphilones C (**61**) exhibited antifungal potential in the range of 67% to 78.6% as against carbendazim, which exhibited an inhibition in the range of 26.4% to 90.7% [61].

One strain of many compounds (OSMAC) was employed on *Preussia isomera*, which was an endophytic fungus of *Panax notoginseng*, leading to the isolation of a known polyketide setosol (**62**) (Figure 3c). It exhibited antifungal activity against *Gibberella saubinetii* with a MIC of 50 μg/mL [62].

## 5. Xanthones

Xanthones are a group of aromatics oxygenated heterocyclic compounds characterized by a dibenzo-γ-pyrone scaffold in their framework [63]. They generally have a tricyclic structure wherein two benzene rings (A and B) are connected via a central pyrone ring. Several derivatives of xanthones have been expressed by cultivable endophytic fungi and exhibit varieties of bioactivities, including antifungal, antioxidant, anti-inflammatory, and anti-cancer properties [64,65,66].

**Figure 4:**
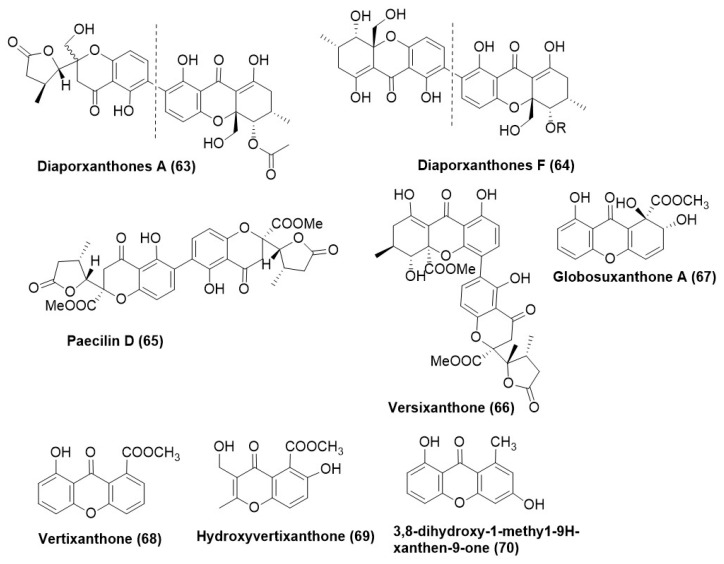
Anti-fungal secondary metabolites produced by fungal endophytes belonging to Xanthone class (**63**–**70**).

Gentisin (1,7-dihydroxy-3-methoxy xanthone) was the first natural xanthone isolated from *Gentiana lutea*, and tajixanthone (prenylxanthine derivative) was isolated from the mycelium of *Aspergillus stellatus* in 1970 [67,68]. Xanthones exert their antifungal action by interfering with the sterol, primarily ergosterol biosynthesis, via targeting enzymes such as squalene epoxidase or 14α-demethylase. Inhibition of these enzymes results in defective cell membrane formation, resulting in the death of the fungal cell [69]. This aspect has been duly validated by the lower content of ergosterols in whole cells of *C. albicans*, *Cryptococcus neoformans*, *Aspergillus fumigatus*, and *Trichophyton mentagrophytes* in the presence of Xanthones [70]. Ergosterol is the prominent sterol in the fungal cell membrane and is responsible for maintaining cellular integrity function and normal growth [71,72,73]. Thus, most antifungal agents act on ergosterol biosynthesis (azoles/allylamines) or ergosterol (amphotericin B). Two xanthone dimers, diaporxanthones A (**63**) and diaporxanthones F (**64**) (Figure 4), were isolated from the culture filtrate of *Diaporthe goulteri* L17, which exists as an endophyte in fruits of salt tolerant *Vitex trifolia*. Diaporxanthones A and B (**63**) and (**64**) exhibited a MIC of 10 µg/mL and 2.5 µg/mL against *Nectria* sp. and *Colletotrichum* sp., respectively. In the in vivo assay, compound (**63**) prevented infection development in the fruit of *V. trifolia* to a great extent [74]. Endophytic *Talaromyces stipitatus* DgCr2 2.1b recovered from the roots of *Duguetia stelechantha* growing in the campus of Universidade Federal do Amazonas, Manaus City, Amazonas State, Brazil, was found to produce Paecilin D (**65**) and versixanthone (**66**) (Figure 4). Versixanthone (**66**) was active against *C. albicans*, *C. tropicalis* with MIC 31.3 µg/mL while Paecilin D (**65**) exhibited a MIC of 15.6 µg/mL for *Candida albicans* and *Candida tropicalis* [36]. *Paraconiothyrium* sp. YM 311593, existing as an endophyte in the fruits of *Azadirachta indica*, was found to produce globosuxanthone A (**67**), vertixanthone (**68**), hydroxyvertixanthone (**69**), 3,8-dihydroxy-1-methy1-9H-xanthen-9-one (**70**) (Figure 4). The antifungal test panel primarily comprised of plant pathogens viz., *Botrytis cineria*, *Fusarium solani* and *P. oryzae*. Globosuxanthone A (**69**) exhibited a MIC in the range of 4–16 µg/mL against the test panel fungi. Moderate activity of Vertixanthone (**68**) was observed against *F. graminearum*, *F. solani*, and *P. oryzae* with MICs ranging from 16–64 μg/mL, respectively. In the case of hydroxyvertixanthone (**69**), *B. cinerea* exhibited a MIC of 64 μg/mL, while *Fusarium graminearum* displayed a MIC of 8 µg/mL. The compound (**70**) exhibited a higher MIC of 128 µg/mL for *P. oryzae* [75].

## 6. Terpenes and Terpenoids

Terpenes generally comprise isoprene units (C_5_H_8_), arranged in numerous ways, leading to the generation of a wide array of chemical structures with equally diverse bioactivities. They have been classified into six groups, namely- Monoterpenes (C_10_H_16_), Sesquiterpenes (C_15_H_24_), Diterpenes (C_20_H_32_), Triterpenes (C_30_H_48_), Tetraterpenes (C_40_H_64_) and Polyterpenes. There is more than one mechanism by which the terpenes exert their antifungal action. Terpenes generally disrupt the cell membrane as they integrate into the fungus cell membranes due to their lipophilic nature [76]. This leads to disruption of the cell membrane integrity, which leads to cell death due to membrane leakage. Yet another mechanism is the inhibition of enzymes involved in the biosynthesis of ergosterol, which is the integral component of fungal cell membranes. Some terpenes induce the production of reactive oxygen species (ROS), which eventually cause oxidative damage to the cellular components, leading to the death of the fungus [77,78].

**Figure 5:**
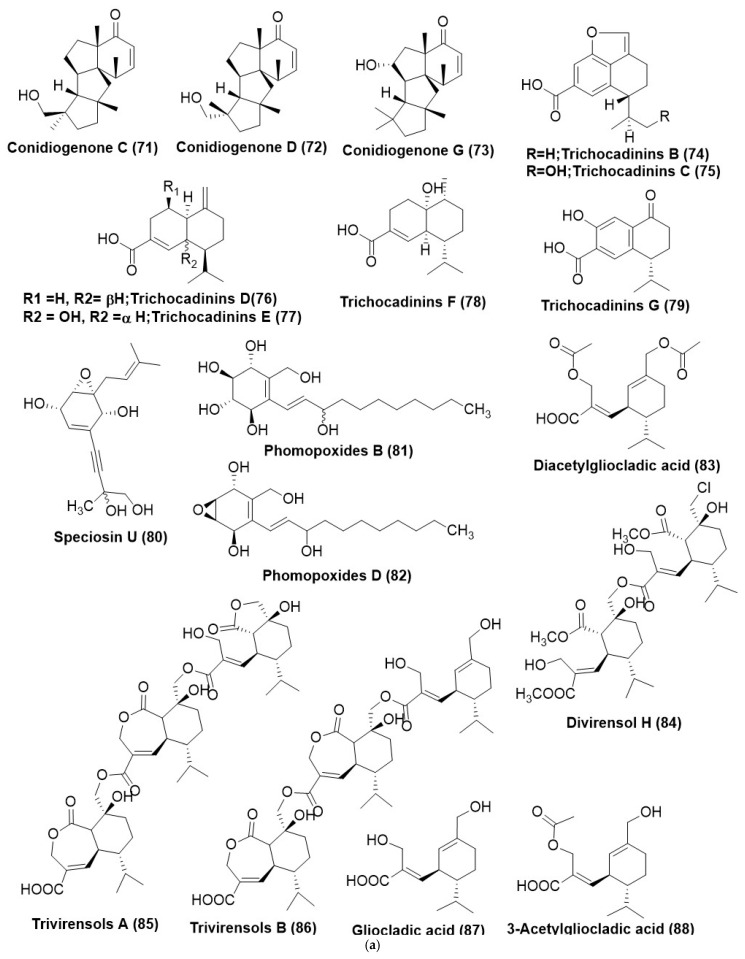

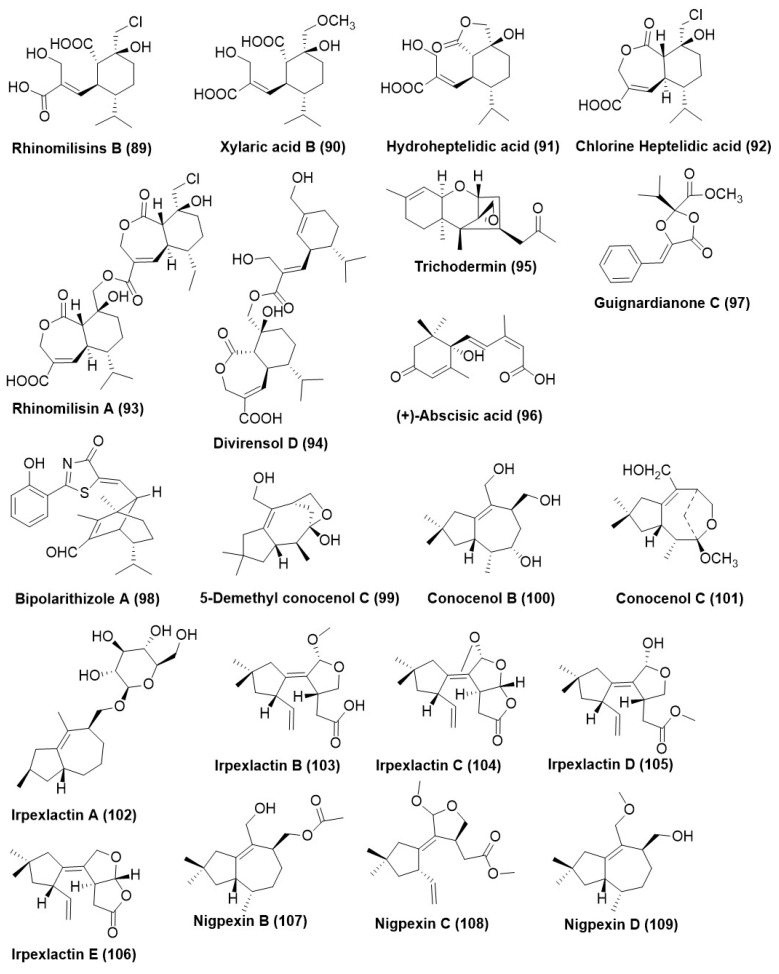

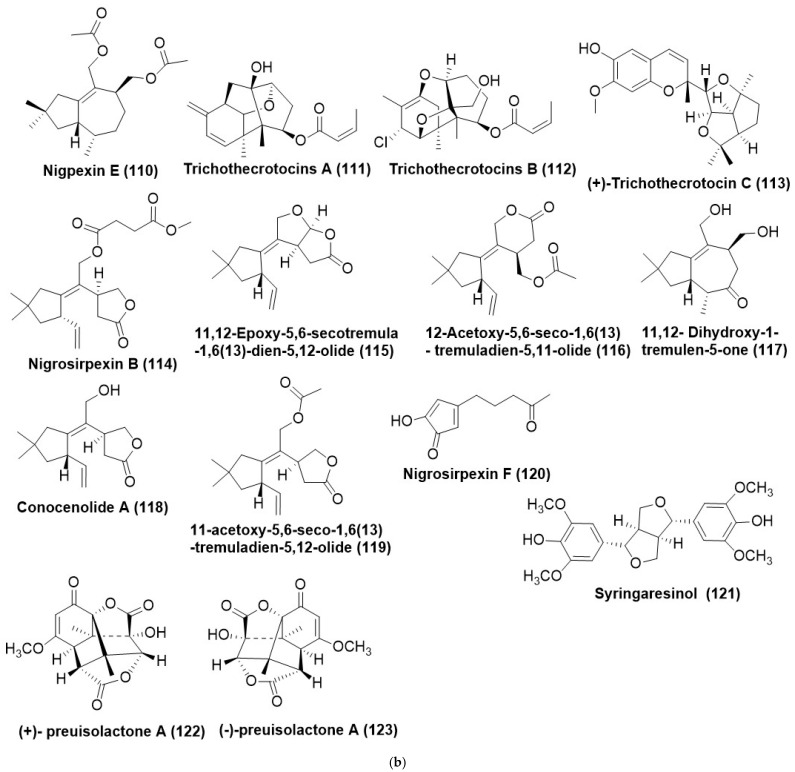
(**a**). Antifungal terpenes and terpenoids isolated from endophytic fungi (**71**–**88**); (**b**) Antifungal terpenes and terpenoids isolated from endophytic fungi (**89**–**123**).

*Leptosphaeria* sp. XL026 residing in the leaves of *Panax notoginseng* was found to produce known diterpenes conidiogenone C (**71**), conidiogenone D (**72**), and conidiogenone G (**73**) (Figure 5a). Compounds (**71**) and (**73**) displayed average antifungal activity against *Rhizoctonia cerealis*, while compound (**72**) exhibited a MIC of 12.5 mg/mL against *Verticillium dahlia* [79]. Trichocadinins B−G (**74**–**79**) (Figure 5a), six unconventional cadinene type sesquiterpene derivatives, were isolated from an endophytic fungus *Trichoderma virens* QA-8, residing inside the inner tissue of plant *Artemisia argyi*. Compound (**74**) exhibited inhibitory activity against *A. solani* QDAU-14, *Bipolaris sorokiniana* QDAU-7, *Ceratobasidium cornigerum* QDAU-8, *Coniothyrium gloeosporioides* Penz. QDAU-9, *Fusarium graminearum* QDAU-10, *F. oxysporum* f. sp. *cucumebrium* QDAU-16, *F. oxysporum* f. sp. *momordicae* QDAU-17, *F. oxysporum* f. sp. *radices lycopersici* QDAU-5, *F. solani* QDAU-15, *Glomerella cingulata* QDAU-2, *Penicillium digitatum* QDAU-11, *P. piricola* Nose QDAU-12, and *Valsa mali* QDAU-13 with MIC in the range of 1–64 μg/mL. At the same time, positive control amphotericin B was found active against the same set of fungi with MIC in the range of 0.5–8 μg/mL. Compounds (**74**–**79**) exhibited antifungal activity with MIC values ranging from 1 to 64 μg/mL against *F. oxysporum* f. sp. *cucumebrium* [80].

Speciosin U (**80**) (Figure 5a) is an oxygenated cyclohexanoid that was isolated and characterized from the fungus belonging to *Saccharicola* sp., existing as an endophyte of *Eugenia jambolana* was collected in Araraquara city, São Paulo State, Brazil. Compound (**80**) exhibited weak antifungal activity against *C. cladosporioides* [81]. New polyoxygenated cyclohexenoids, namely, phomopoxides B (**81**) and D (**82**) (Figure 5a), were characterized from *Phomopsis* sp. YE3250 is a fungus residing inside the stems of *Paeonia delavayi* in China. An intermediate antifungal activity was exhibited by phomopoxides D (**82**) against *Aspergillus niger* and *Candida albicans* with a MIC value of 64 μg/mL. Test panel comprising *Histoplasma compactum*, *Aspergillus niger*, and *Candida alblicans* exhibited a MIC value of 64 μg/mL, 64 μg/mL, and 32 μg/mL, respectively [82].

Diacetylgliocladic acid (**83**), a novel mono-sesquiterpene, divirensol H (**84**) (Figure 5a), a dimeric sesquiterpene, along with and two novel trimeric sesquiterpene trivirensols A (**85**), and B (**86**), and eight known congeners, gliocladic acid (**87**), 3-acetylgliocladic acid (**88**), rhinomilisin B (**89**), xylaric acid B (**90**), hydroheptelidic acid (**91**), chlorine heptelidic acid (**92**), rhinomilisin A (**93**) and divirensol D (**94**) (Figure 5a), were extracted and identified from *Trichoderma virens* FY06, an endophytic fungus from *Litchi chinensis* stored at the College of Materials and Energy, South China Agricultural University. Compounds (**83**–**94**) displayed antifungal activity against *F. oxysporum*, *Colletotrichum gloeosporioides*, *C. musae*, *Penicillium italicum*, and *Fusarium graminearum* with MICs in the range of 6.5 to 200 μg/mL. Positive control triadimefon displayed antifungal activity against *F. oxysporum*, *C. gloeosporioides*, *C. musae*, *P. italicum*, and *F. graminearum* with MICs in the range of 50 to 150 μg/mL [83].

Trichodermin (**95**) (Figure 5a) was purified from *Trichoderma koningiopsis* VM115 and isolated from an endophytic fungus associated with *Vinca* sp. from Iran. Trichodermin (**95**) displayed potent antifungal activity against *Pyricularia oryzae*, *Aspergillus fumigatus*, and *Botrytis cinerea* with MICs of 31.25 μg/mL each. Positive control ketoconazole displayed antifungal activity with MIC values against *P. oryzae*, *A. fumigatus*, and *B. cinerea* with MICs of 62.5, 31.2, and 62.5 μg /mL [14]. Sesquiterpene phytohormone (+)-abscisic acid (**96**) (Figure 5b) produced by the endophytic fungus *Fusarium verticillioides* strain WF18 residing within the leaves of *Mentha piperita* exhibited antifungal activity against *Aspergillus austroafricanus* strain EIS1, *Aspergillus versicolor* strain EIS2, *Phoma fungicola* strain AT100, *Aspergillus tubingensis* strain AN103, and *C. albicans* ATCC 90029 with MIC in range of 0.4–0.7 μg /mL [40].

A dioxolanone derivatives guignardianone C (**97**) (Figure 5b) was isolated from *Phyllosticta* sp. WGHL2, the endophytic fungus residing inside the leaves of *Osmanthus fragrans*, was collected from Nanjing Agricultural University, China. Compound (**97**) displayed broad-spectrum antifungal activities against *Rhizoctonia solani*, *Fusarium graminearum*, and *B. cinerea* with inhibition ratios of 48.43%, 40.98%, and 49.53% at 50 mg/mL, respectively. Moreover, compound (**97**) showed a moderate protective effect against *B. cinerea* in vivo at 200 mg/mL and exhibited effective inhibition on the spore germination of *B. cinerea* [84].

Bipolarithizole A (**98**) (Figure 5b), an unprecedented phenylthiazole-sativene sesquiterpenoid hybrid, was produced by *Bipolaris eleusines*, an endophyte of *Solanum tuberosum*. The absolute configuration of Bipolarithizole A (**98**) was established by spectroscopic methods, quantum chemical computational approach, and X-ray diffraction studies. Compound (**96**) exhibited a MIC of 16 μg /mL against *Rhizoctonia solani* [85].

Endophytic *Irpex lacteus* and *Nigrospora oryzae*, when co-cultured, produced two new tremulane sesquiterpenes, 5-demethyl conocenol C (**99**) (Figure 5b), nigrosirpexin A (**57**) (Figure 3b) and known sesquiterpenes conocenol B (**100**) and conocenol C (**101**) (Figure 5b). The compound (**100**) exhibited a MIC in the range of 1–512 μg/mL against *Colletotrichum gloeosporioides*, *Cladosporium tenusissimum*, *Phoma herbarum*, and *Didymella glomerata*. Nigrosirpexin A (**57**) against the test panel of fungi exhibited a MIC in the range of 16–512 μg/mL, while conocenol B (**100**) had a MIC in the range of 8–512 μg/mL. Conocenol C (**101**) exhibited MIC ranging from 4 μg/mL to 512 μg/mL. The positive control, Nystatin, however, exhibited a wide spectrum of MIC against the test panel fungi, beginning from 4 μg/mL to 512 μg/mL [86].

Endophyte *Irpex lacteus* cultured in host *Gastrodia elata* yielded five new tremulane sesquiterpenes irpexlactin A-E (**102**–**106**) (Figure 5b). Compounds (**104**–**108**) were found active against *P. polonicum*, *T. atroviride*, *P. subsingeri*, and *Armillaria* sp. with MIC values in the range of 4–64 μg/mL. Compounds (**102**), (**103**), and (**105**) were active against *I. lacteus* with MIC values of 32–64 μg/mL. Positive control nystatin displayed antifungal activity against *P. polonicum T. atroviride*, *P. subsingeri*, and *I. lacteus* with MIC values in the range of 4–16 μg/mL [87].

New template sesquiterpenes analogous to digoxin, namely nigpexin B-D (**107**–**109**) and nigpexin E (**110**) (Figure 5b), were isolated from the co-culture of three microorganisms viz. a phytopathogen and endophyte and entomopathogen respectively identified as *Irpex lacteus*, *Nigrospora oryzae*, and *B. bassiana*. MIC of the compounds, nigpexin B-D (**107**–**109**) against *Irpex lacteus* was found to be <8 µg/m L. Co-culture of endophytic *Irpex lacteus* and *Nigrospora oryzae* afforded the production of two new tremulane sesquiterpenes 5-Demethyl conocenol C (**99**), nigrosirpexin A (**57**). The compound (**107**) from *I. lacteus* showed antifungal activity against *N. oryzae* with MIC at 32 μg/mL. *Trichothecium crotocinigenum*, an endophytic fungus residing in *Solanum tuberosum* from China was found to produce two trichothecene sesquiterpenoids, trichothecrotocin A (**111**) and trichothecrotocin B (**112**), and a merosesquiterpenoid racemate, (±)-trichothecrotocin C (**113**) (Figure 5b). A new 6/6-5/5/5 fused ring system was found in the compounds (**111**) and (**112**). Compound (**113**) displayed antifungal activity against *Phytophthora infestans*, *A. solani*, *R. solani*, and *F. oxysporum* in the range of 8–128 µg/mL. Positive control hygromycin displayed antifungal activity against *P. infestans*, *A. solani*, *R. solani*, and *F. oxysporum* in the 4–64 µg/mL range. Compound (**113**) displayed antifungal activity against *A. solani* and *F. oxysporum* with MIC values of 16 and 32 µg/mL, respectively. Compound (**112**) displayed antifungal activity against *A. solani* and *F. oxysporum* with MIC values of 32 and 16 µg/mL, respectively [88].

Tremulane sesquiterpenoids like Nigrosirpexin B (**114**), 11,12-epoxy-5,6-secotremula-1,6(13)-dien-5,12-olide (**115**), 12-acetoxy-5,6-seco-1,6(13)-tremuladien-5,11-olide (**116**), 11,12- dihydroxy-1-tremulen-5-one (**117**), conocenolide A (**118**), and 11-acetoxy-5,6-seco-1,6(13)-tremuladien-5,12-olide (**119**) (Figure 5b), conocenol B (**100**), were identified from the co-culture of *Nigrospora oryzae* and *Irpex lacteus* (phytopathogen-endophyte combination). Nigrosirpexin F (**120**) and syringaresinol (**121**) (Figure 5b) by another co-culturing combination comprised of the endophytic fungi with their host plant, were identified from co-culture of endophytic fungi and the host plant, namely *N. oryzae*, *I. lacteus* and *Dendrobium officinale* (Host plant). Selective antifungal activity was reported by the compounds (**114**–**119**) and (**100**) against *N. oryzae* with MIC ranging from of 1–256 μg/mL. Compounds (**114**) and (**118**) presented notable anti-phytopathogenic activity that was equivalent to that of nystatin. Compounds (**115**), (**120**), and (**121**) (Figure 5b) were found to have an inhibitory activity against the phytopathogen *N. oryzae* with a MIC of 256, 64, and 16 μg/mL, respectively. However, the potent antifungal activity of the compound (**120**) was observed against *Irpex lacteus* with a MIC of 8 μg/mL [89].

A pair of enantiomeric nor-sesquiterpenoids, (+)- and (−)-preuisolactone A (I and II) (**122**) and (**123**) (Figure 5b) possessing a unique tricyclo [4.4.01,6.02,8] decane scaffold was isolated from *Preussia isomera*. Compounds (+)-I and (−)-II (**122**) and (**123**) are rare naturally occurring sesquiterpenoid enantiomers. (±)-Preuisolactone A (**122**) and (**123**) have poor antifungal activity against *Alternaria alternata* with a MIC value of 50.04 μg/mL as compared to Ketoconazole with a MIC of 1.382 μg/mL [90]. *Preussia* species generally produce an array of antifungal compounds, which is mainly attributed to interference competition that they generally face in the dung, i.e., they are coprophilic in nature, and these antifungal secondary metabolites produced by them are the drivers of the colonial succession [90].

## 7. Other Miscellaneous Compounds

### 7.1. Acids and Their Derivatives

**Figure 6:**
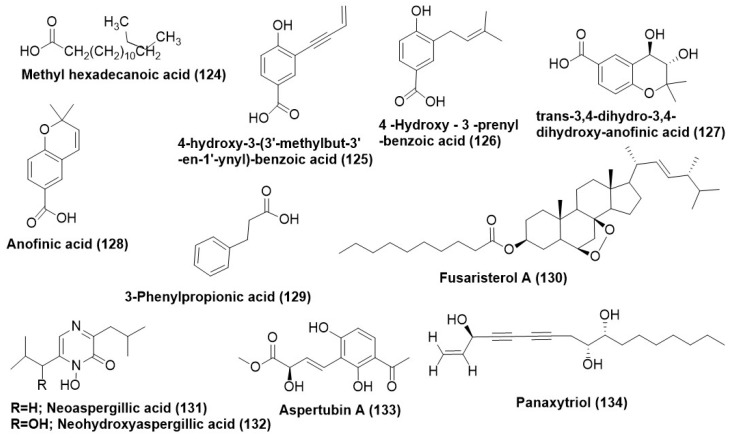
Acid and acid derivatives produced by endophytic fungi exhibiting anti-fungal potential (**124**–**134**).

Methylhexadecanoic acid (**124**) (Figure 6) was purified from *Macrophomina phaseolina* and demonstrated antimycotic activity towards *F. oxysporum* and *S. sclerotium* with an IC_50_ value of 0.662 µg/mL and 1.002 µg/mL respectively. 4-hydroxy-3-(3′-methylbut-3′-en-1′-ynyl)-benzoic acid (**125**), 4-hydroxy-3-prenyl-benzoic acid (**126**), and trans-3,4-dihydro-3,4-dihydroxy-anofinic acid (**127**) (Figure 6) were also isolated from endophytic *Saccharicola* sp. In a TLC bioautographic assay, compounds (**125**) and (**126**) showed significant antifungal activity against *C. phaerospermum* at 25 μg and 5 μg, respectively. In comparison, Nystatin (positive control) inhibited the fungal growth at a concentration of 1.0 μg [73]. 4-hydroxy-3-prenyl-benzoic acid (**126**) and anofinic acid (**128**) (Figure 6) were isolated from *Drechslera* sp.**,** an endophytic fungus associated with the roots of ryegrass (*Lollium* sp.). The compounds (**126**) and (**128**) in the TLC direct bioautography exhibited inhibition halos of 20 mm (±1) each against *Fusarium tucumaniae*, while the positive controls benomyl and carbendazim exhibited inhibition zones of 20 mm and 22 mm, respectively [91].

3-phenylpropionic acid (**129**) (Figure 6) isolated from endophytic *Cladosporium cladosporioides*, exhibited a MIC of 3.9 µg/mL and 7.8 µg/mL against *Fusarium solani* and *Aspergillus flavus*, respectively. However, the positive control Amphotericin B exhibited a significantly lower MIC of 0.49 µg/mL. A new ergosterol derivative, namely, fusaristerol A (22E,24R-5b,8b-epidioxyer-gosta-22-en-3b-yl decanoate) (**130**) (Figure 6), was purified from endophytic fungus *Fusarium* sp. isolated from the root of *Mentha longifolia* L. Compound (**130**) found active toward *C. albicans* with MIC value of 8.3 µg/disk while positive control clotrimazole displayed antifungal activity with MIC value of 5.1 µg/disk [92].

Neoaspergillic acid (**131**) and neohydroxyaspergillic acid (**132**) (Figure 6) are hydroxamic acids that were purified from *Aspergillus* sp. SPH2 recovered from the stem of *Bethencourt iapalmensis*, an endemic plant in Abona, Spain. Compound (**131**) displayed antifungal activity with EC_50_ values of 70, 10, and 40 µg/mL, respectively, against *F. oxysporum*, *A. alternata*, and *B. cinerea *in mycelial growth inhibition assay. Compound (**132**) displayed antifungal activity with EC_50_ values of 200 µg/mL respectively against *A. Alternata in* mycelial growth inhibition assay [9].

Aspertubin A (**133**) (Figure 6) is a new globoscinic acid derivative which along with a new compound panaxytriol (**134**) (Figure 6) was isolated from the co-culture of red ginseng and *Aspergillus tubingensis* S1120. Compounds (**133**) and (**134**) exhibited antifungal activity with MIC of 8 µg/mL against *Aspergillus tubingensis* [93].

### 7.2. Furans and Their Derivatives

**Figure 7:**
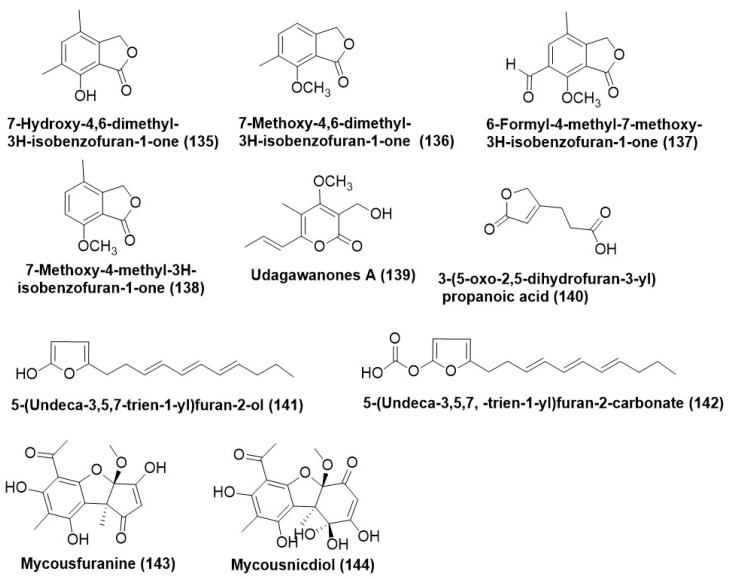
Furans and their derivatives with antifungal activity isolated from endophytic fungi.

Known isobenzofuranones, 7-hydroxy-4,6-dimethyl-3H-isobenzofuran-1-one (**135**), 7-methoxy-4,6-dimethyl-3H-isobenzofuran-1-one (**136**), 6-formyl-4-methyl-7-methoxy-3H-isobenzofuran-1-one (**137**), and 7-methoxy-4-methyl-3H-isobenzofuran-1-one (**138**) (Figure 7) were purified from *Nodulisporium* sp. GS4d2II1a (anamorph of *Hypoxylon anthochroum* GS4d2II1a or Gseg1) isolated from the leaves of *Gliricidia sepium* growing in Mexico. Compounds (**135**–**138**) were found to be active against *F. oxysporum*, *Alternaria alternata*, *Pythium aphanidermatum*, and *Phytophthora capsici*, with IC_50_ values in the range of 0.00011 µg/mL to 0.00021 µg/mL. The possible mechanism of activity of the compounds (**135**) and (**136**) is via cell membrane damage leading to electrolyte leakage. Morphological changes in the mycelia of the plant pathogens were observed with the use of compounds (**135**–**138**) as antifungals [94].

A new α-pyrone derivative, udagawanone A (**139**) (Figure 7), was purified from *Neurospora udagawae*, an endophyte associated with the plant *Quercus macranthera* collected from the Kaleybar region in northwestern Iran. Compound udagawanone A (**139**) exhibited moderate antifungal activity against *Rhodotorula glutinis* (MIC = 66 μg/mL) [95]. *Aspergillus tubingensis*, existing as an endophyte of *Decaisnea insignis* growing in Shaanxi Province, China, was the source of a new furan derivative named 3-(5-oxo-2,5-dihydrofuran-3-yl) propanoic acid (**140**) (Figure 7). Compound (**140**) was evaluated on a test panel of plant pathogenic fungi comprising *A. alternata* BNCC 341716, *S. sclerotiorum* BNCC 122299, *Botrytis cinerea* BNCC 338228, *Phytophthora capsici* BNCC 339721 and exhibited a MIC range of 16–64 µg/mL. Carbendazim served as a positive control and exhibited a MIC in the range of 32–64 µg/mL [96].

5-(undeca-3′,5′,7′-trien-1′-yl) furan-2-ol (**141**) and 5-(undeca-3′,5′,7′-trien-1′-yl) furan-2-carbonate (**142**) (Figure 7) are the two new alkylated furan derivatives which were identified from *Emericella* sp. XL029, residing in the leaves of *Panax notoginseng*. Compound (**141**), during in vitro evaluation against a phytopathogenic test panel, exhibited a MIC in the range of 25 to 3.1 μg/mL, while compound (**142**) in a phytopathogenic panel displayed higher MIC values ranging between 50–12.5 μg/mL [97].

Endophytic *Mycosphaerella* species UFMCB 2032 recovered from the leaves of *Eugenia bimarginata* located in Brazilian savanna produces two novel usnic acid derivatives Mycousfuranine (**143**) and mycousnicdiol (**144**) (Figure 7)**.** Both compounds (**143**) and (1**44**) exhibited average antifungal activity against *Cryptococcus neoformans* and *C. gattii* with a MIC of 50 μg/mL and 250 μg/mL respectively [98].

### 7.3. Benzene and Derivatives

**Figure 8:**
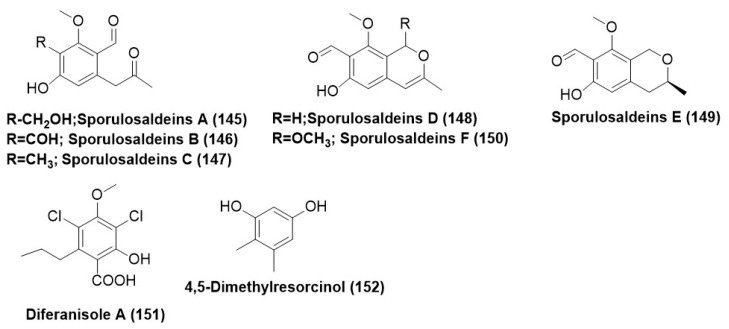
Benzene and benzene derivatives with antifungal properties from endophytic (**145**–**152**).

*Paraphaeosphaeria* sp. F03, existing as an endophyte in the leaves of *Paepalanthus planifolius*, was found to produce three Sporulosaldeins A–C (**145**–**147**), new benzaldehyde derivatives, and sporulosaldeins D–F (**148**–**150**) (Figure 8). On an antifungal test panel comprising of different *Candida species* (*C. albicans* ATCC 10231, *C. tropicalis* ATCC 750, *C. krusei* ATCC 6258, *C. glabrata* ATCC 2001, and *C. parapsilosis* ATCC 22019. These compounds (**145**–**150**) exhibited a MIC range of 7–250 µg/mL. MIC of 7.8 µg/mL was exhibited by compound (**149**) against *C. glabrata*, *C. tropicalis*, and *C. krusei*. MIC of compound (**148**) was found to be 15.6 µg/mL for *Candida parapsilosis*, while for fluconazole-resistant *Candida albicans*, it exhibited a MIC of 62.5 µg/mL [99].

Compound diferanisole A (**151**) and 4,5-dimethylresorcinol (**152**) (Figure 8) were identified from *Chaetomium* sp. HQ-1 is associated with the plant *Astragalus chinensis* from Shandong Province in China. Compounds diferanisole A (**151**) and 4,5-dimethylresorcinol (**152**) showed potent inhibition activity against *S. rolfsii*, with IC_50_ values less than 16 and 32 µg/mL, respectively [100].

### 7.4. Heterocyclic Compounds

**Figure 9:**
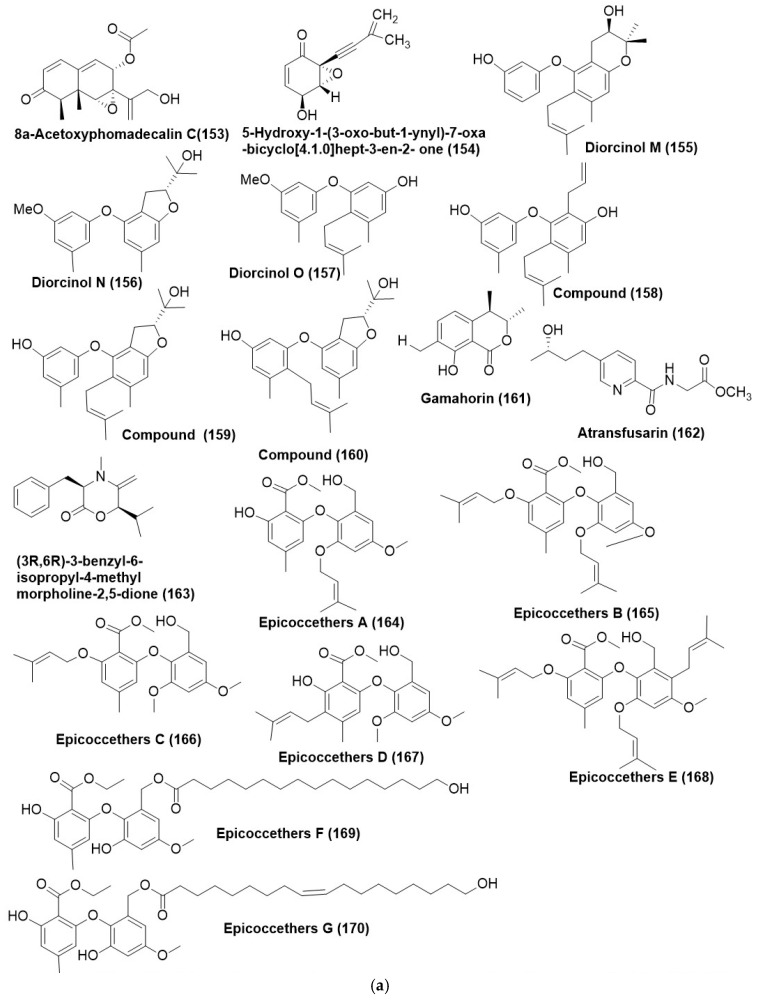

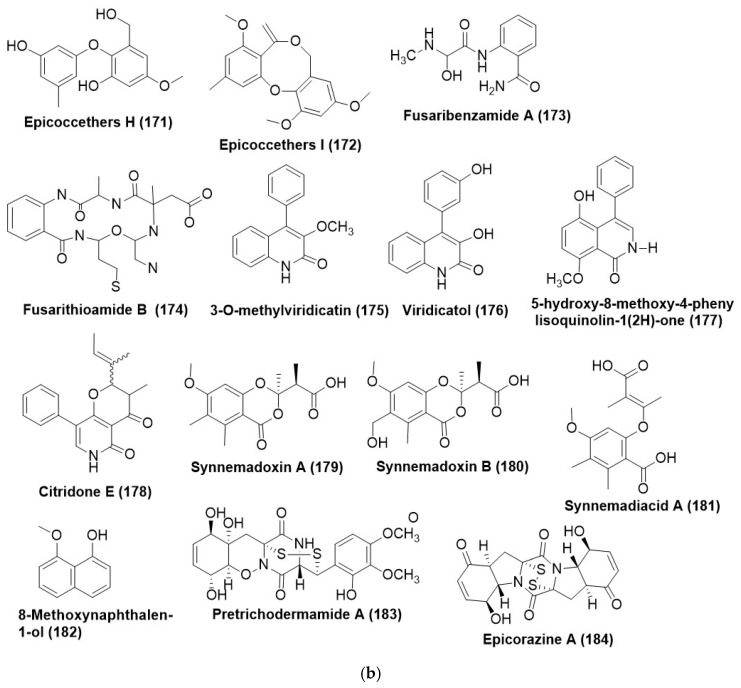
(**a**) Heterocyclic compounds with antifungal activity produced by fungal endophytes (**153**–**170**); (**b**). Antifungal heterocyclic compounds produced by endophytic fungi (**171**–**184**).

8α-acetoxyphomadecalin C (**153**) (Figure 9a) was isolated and characterized from an endophytic fungus, *Microdiplodia* sp. WGHS5 is associated with the leaves of *Osmanthus* sp., which were collected from Nanjing City, Jiangsu Province, China. Compound (**153**) showed inhibition against *B. cinerea* and *Fusarium graminearum* with inhibitory ratios of 81.8% and 59.6%, respectively, at the concentration of 100 μg/mL [101]. 5-hydroxy-1-(3-oxo-but-1-ynyl)-7-oxa-bicyclo [4.1.0]hept-3-en-2-one (**154**) (Figure 9a) was isolated from endophytic *Drechslera* sp. strain 678, existing within the roots of *Neurachne alopecuroidea*, an Australian native grass. Compound (**154**) effectively controlled the plant pathogen *S. sclerotiorum* at 10 and 100 µg/mL, while the lowest concentration (1 µg/mL) was less effective against *S. sclerotiorum*. Compound (**154**) also inhibited another plant pathogen, *B. cinerea*, at the highest concentration (100 µg/mL) in a dose-dependent manner [42].

Three new prenylated diphenyl ethers, namely diorcinol M-O (**155**–**157**) and previously described compounds (**158**–**160**) (Figure 9a), were purified from *Arthrinium arundinis* TE-3, an endophytic fungus residing inside the leaves of tobacco collected from Wangcheng Slope, Enshi, Hubei, China. Compound (**155**) was found active against *Alternaria alternata*, *Glomerella cingulate*, and *Mucor hiemalis* with MIC values of 64, 32, and 8 μg/mL, respectively. Compound (**156**) displayed antifungal activity against *A. alternate* and *M. hiemalis* with MIC values of 32 and 4 μg/mL, respectively. Compound (**157**) displayed antifungal activity against *A. alternata*, *Cochliobolus heterostrophus*, *Gaeumannomyces graminis*, and *M. hiemalis with* MIC in the 16–64 μg/mL range. Compound (**158**) was found active against *C. heterostrophus*, *Glomerella graminis*, *M. hiemalis*, and *Thielaviopsis basicola* with MIC in the range of 16–64 μg/mL. Compound (**159**) was found active against *A. alternata*, *G. graminis*, *G. cingulate*, *M. hiemalis*, and *T. basicola* with MIC in the 8–64 μg/mL range. Compound (**160**) was found active against *A. alternata* and *G. cingulate* with MIC in the 8- and 16 μg/mL range. Positive control prochloraz displayed antifungal activity with MIC of 8, 8, 8, 16, 8 μg/mL, and Gamahorin (**161**) (Figure 9a) was isolated from the endophytic fungus *Pestalotiopsis microspora* SC3082 recovered from *Scaevola taccada* (Gaertn.) Roxb [102]. Compound (**161**) displayed an average antifungal activity with a MIC value of 12.5 μg/mL against *C. albicans* ATCC 10321 [103].

A new fusaric acid derivative, atransfusarin (**162**) and (3R,6R)-3-benzyl-6-isopropyl-4-methylmorpholine-2,5-dione (**163**) (Figure 9a), were characterized from *Alternaria atrans* MP-7 an endophyte, residing inside the medicinal plant *Psidium guajava* collected from Nanning city, Guangxi Province of China. Compound (**163**) found active against *A. solani*, *Colletotrichum gloeosporioides*, and *Phyricularia grisea* with MIC of 1.63 µg/mL and against *Botrytis cinerea* with MIC of 6.53 µg/mL. Positive control carbendazim displayed antifungal activity against *B. cinerea*, *A. solani*, *C. gloeosporioides*, and *P. grisea* with MIC of 6.53, 3.27, 13.06, and 13.06 µg/mL, respectively. Compound (**162**) displayed poor activity against *B. cinerea* and *A. solani* (MIC = 13.32 µg/mL) [104].

Nine new diphenyl ethers, epicoccethers A-F (**164**–**170**) (Figure 9a), and epicoccethers G-I (**170**–**172**) (Figure 9b) purified from the *Epicoccum sorghinum* L28, an endophytic fungus of *Myoporum bontioides* using IEMAHC” (Induction of Endophyte Metabolism by Adding Host Components) Guaiol, an ingredient of *M. bontioides*, was used as an inducer. Compounds (**168**–**170**) were products generated by induction of guaiol. Compounds (**163**–**171**) displayed average or good inhibitory activities (MICs 25–200 μg/mL) towards *Colletotrichum musae*, and *Fusarium oxysporum*, except that (**164**) and (**171**) were inactive to *C. musae*. Positive control triadimefon displayed inhibitory activities with MIC of 80 and 100 μg/mL against *C. musae*, and *F. oxysporum* [105].

A new aminobenzamide derivative, fusaribenzamide A (**173**) (Figure 9b), was isolated from *Fusarium* sp., an endophyte residing inside the root of *Mentha longifolia* collected from Saudi Arabia. Compound (**173**) displayed strong antifungal activity against *C. albicans* with MIC value of 11.9 mg/disk (Positive control nystatin, MIC 4.9 mg/disk) [105].

Another new aminobenzamide derivative, fusarithioamide B (**174**) (Figure 9b), was purified from *Fusarium chlamydosporium*, an endophyte of the leaves of *Anvillea garcinii*. Fusarithioamide B (**174**) was selectively found active against *C. albicans* with a MIC value of 1900 µg/mL and inhibition zone diameter (IZD) of 14.5 mm, while positive control clotrimazole displayed antifungal activity with MIC of 2800 µg/mL and IZD 17.9 mm. Compound (**174**) displayed moderate activity against *G. candidum* with a MIC value of 6900 µg/mL and IZD 28.9 mm [106]. 3-O-methylviridicatin (**175**), viridicatol (**176**) are quinoline alkaloids, a novel isoquinoline alkaloid, 5-hydroxy-8-methoxy-4-pheny isoquinoline-1 (2H)-one (**177**) (Figure 9b) produced by the endophytic fungus *Penicillin* sp. R22, which exists as an endophyte in *Nerium indicum*. 3-O-methylviridicatin (**175**) exhibited a MIC of 31.2 μg/mL against *Alternaria brassicae* and *Valsa mali*, while viridicatol (**176**) was active against *Botrytis cinerea* and exhibited MIC as the same value as compound (**175**). Compound (**177**) was specifically active against *B. cinerea* with a MIC value of 31.2 μg/mL [102].

Citridone E (**178**) is an indole alkaloid isolated from endophytic *Penicillium sumatrense* GZWMJZ-313 residing inside the plant *Garcinia multiflora*. Citridone E (**178**) (Figure 9b) exhibited a MIC of 32 μg/mL and >128 μg/mL for *C. glabrata* ATCC2001 and *C. albicans* ATCC 10231, respectively. However, the positive control, ketoconazole, exhibited a MIC of 2 and 4 μg/mL, respectively, for *C. glabrata* ATCC2001 and *C. albicans* ATCC 10231 [50].

Synnemadoxins A and B (**179**) and (**180**) (Figure 9b) possessing rare 1,3-benzodioxin-4-one scaffold and their postulated precursor, synnemadiacid A (**183**), were isolated from *Synnemapestaloides ericacearum* as an endophyte of *Rhododendron groenlandicum* and *Kalmia latifolia* from Canada (southern New Brunswick)**.** Synnemadoxins A-B (**179**–**180**) and synnemadiacid A (**181**) displayed potent inhibitory activity against *M. violaceum* (formerly *Ustilago violacea*), with a MIC of 2.3 μg/mL [107].

Compound 8-methoxynaphthalen-1-ol (**182**) (Figure 9b) was purified from *Diatrype palmicola* MFLUCC 17-0313, an endophytic fungus residing inside the leaves of *Polyscias fruticose* collected from Mae Chan District, Chiang Rai Province, Thailand. Compound (**182**) exhibited an average antifungal activity against *Athelia rolfsii* with a MIC of 250 µg/mL [108].

Known (epi)dithiodiketopiparazines viz. pretrichodermamide A (**183**) and epicorazine A (**184**) (Figure 9b) were characterized from *Trichoderma harzianum* and *Epicoccum nigrum*, endophyte of *Zingiber officinale* collected in Banyumas, Central Java, Indonesia and *Salix* sp., collected in Lago Naki, the Republic of Adygea (North Caucasus), Russia respectively. Compound (**183**) displayed selective antifungal activity against *U. maydis* with a MIC value of 1 mg/mL, while positive control nystatin displayed antifungal activity against a MIC value of 0.02 mg/mL. Compounds (**183**–**184**) exhibited potent antifungal activity against *Ustilago maydis*, with zones of inhibition of 15 and 10 mm at 100 µg/disk, respectively. Meanwhile, nystatin at 10 mg/mL and nourseothricin at 20 mg/mL displayed antifungal activity against *U. maydis* with an inhibition zone of 29 and 14 mm, respectively [109].

### 7.5. Ketones and Their Derivatives

**Figure 10:**
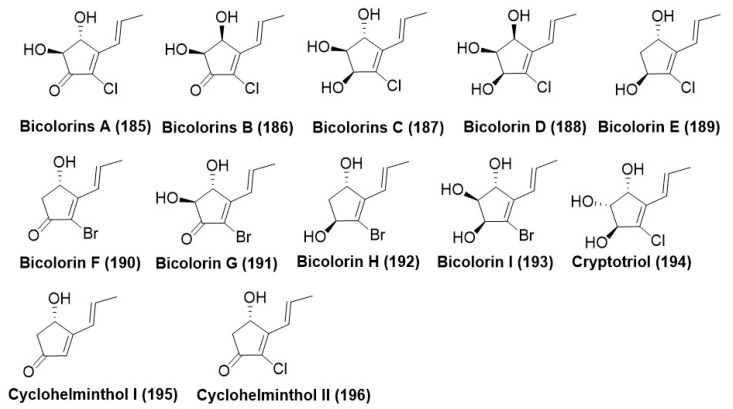
Ketone and ketone derivatives from endophytic fungi with antifungal activity (**185**–**196**).

*Saccharicola bicolor*, an endophyte of *Bergenia purpurascens* growing in the Tibet Autonomous Region, China, produced nine new halogenated cyclopentenones named Bicolorins A-I (**185**–**193**) (Figure 10), along with three previously reported cyclopentenones, cryptotriol (**194**), cyclohelminthol I (**195**), and cyclohelminthol II (**196**) (Figure 10) by using one strain many compounds (OSMAC) strategy. Compounds (**185**–**193**) were found active against phytopathogenic fungus *Uromyces viciae-fabae*, *Pythium dissimile*, *Gibberella zeae*, *Aspergillus niger*, and *Sclerotinia sclerotiorum* with MIC in the range of 6.2–100 μg/mL. Potential antifungal activity of the compounds (**186**) and (**188**) against *P. dissimile* with MIC of 6.2 and 8.5 μg/mL, respectively, compared with the positive control Cycloheximide having a MIC of 8.6 μg/mL. Compound (**188**) also displayed potent antifungal activity against *S. sclerotiorum* with a MIC value of 9.8 μg/mL compared to 11.6 μg/mL of the positive control cycloheximide [110].

### 7.6. Macrocyclic Lactones/Macrolides

**Figure 11:**
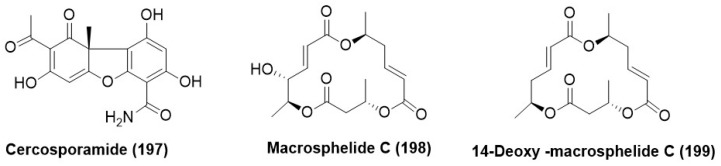
Antifungal macrolides/macrocyclic lactones produced by endophytic fungi (**197**–**199**).

Cercosporamide (**197**) (Figure 11) was purified from *Cadophora orchidicola* ZJLQ336**,** an endophytic fungus from *Kalimeris indica* collected in Long Quan County, Zhejiang Province, China. Cercosporamide (**197**) displayed antifungal activity against *Pestalotia diospyri*, *Botrytis cinerea*, *F. oxysporum*, *Sclerotium rolfsii*, and *Penicillium digitatum* with EC_50_ values of 5.29 × 10^–3^, 0.61, 0.93, 2.89 and 6.7 µg/mL respectively. *S. rolfsii* was one of the main pathogens in *K. indica* [88]. Macrosphelide C (**198**) and 14-deoxy-macrosphelide C (**199**) (Figure 11) were isolated from and purified from *Drechslera* sp., an endophytic fungus associated with the roots of ryegrass (*Lollium* sp.). The compounds (**198**) and (**199**) displayed antifungal activity against the plant pathogen *Fusarium tucumaniae*, with inhibition halos of 20 and 12 and (±1), respectively, by the direct bioautographic method on TLC (Thin layer chromatography) at a concentration level of 20 µg/spot of each assayed compound. Positive controls, carbendazim, and benomyl, showed inhibition zones of 22 and 20 mm, respectively [91].

### 7.7. Peptides and Their Derivatives

**Figure 12:**
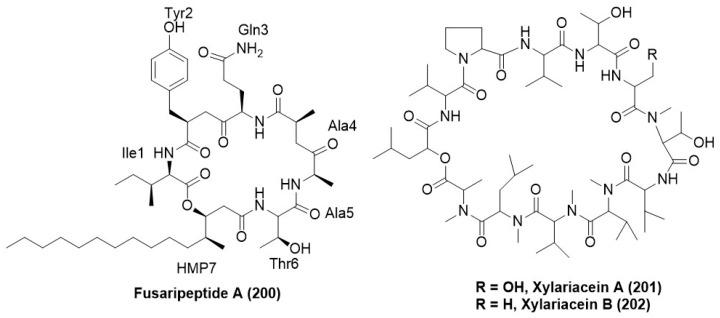
Peptides and their derivatives with antifungal activity isolated from endophytic fungi (**200**–**202**).

A new cyclodepsipeptide, namely fusaripeptide A (**200**) (Figure 12), was identified from the *Fusarium* sp., an endophytic fungus residing inside the roots of *Mentha longifolia* collected from Saudi Arabia. Fusaripeptide A (**200**) displayed strong antifungal activity with IC_50_ values of 0.099, 0.22, 0.171, and 0.126 µg/mL against *C. albicans*, *C. glabrata*, *C. krusei*, and *A. fumigatus* respectively [60]. The cyclic depsipeptide xylariacein A (**201**) and B (**202**) (Figure 12) were purified from the fungus *Xylaria* BSNB-0294, which exists as an endophyte from *Astrocaryum sciophyllum*, inhabiting the pristine forests of French Guyana. *Fusarium oxysporum* f. sp. *ciceris* was inhibited by ethyl acetate extract BSNB-0294 as well as isolated compounds xylariacein A (**201**) and B (**202**) [111].

### 7.8. Prenylated Amino Acids

**Figure 13:**
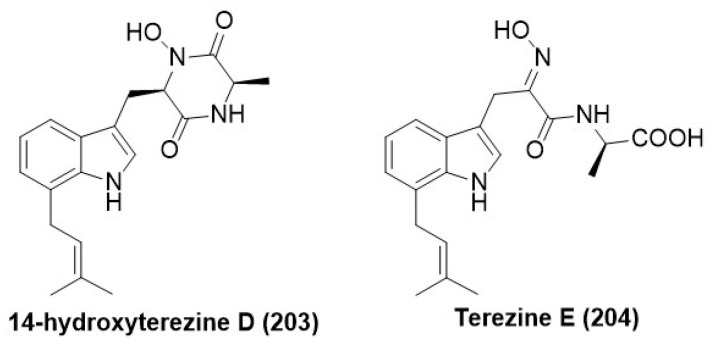
Prenylated amino acids produced by endophytic fungi exhibiting antifungal action (**203**–**204**).

*Mucor* sp., living as an endophyte in the medicinal plant *Centaurea stoebe*, produced two prenylated tryptophan analogs, a previously reported 14-hydroxyterezine D (**203**) and a new terezine E (**204**) (Figure 13). MIC of the compounds (**203**) and (**204**) against *Aspergillus terreus* was found to be 43.6 and 39.7 µg /mL, respectively, whereas the positive control Nystatin exhibited a MIC of 15.63 µg /mL [112].

### 7.9. Quinones

**Figure 14:**
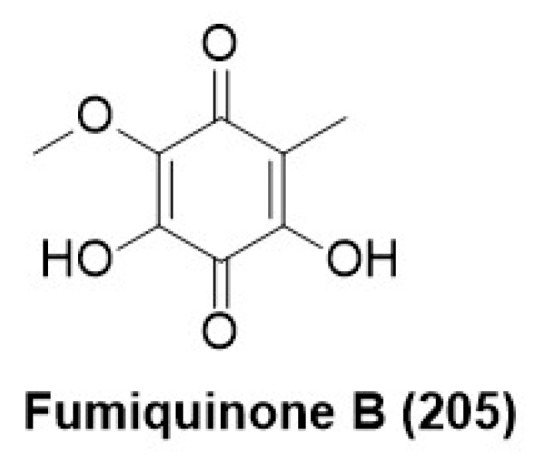
A single quinone with antifungal activity isolated from the endophytic *Neopestalotiopsis* sp. (**205**).

Compound fumiquinone B (**205**) (Figure 14) was purified from *Neopestalotiopsis* sp., an endophyte associated with the plant *Begonia fischeri* collected from Atlantic Rain Forest. Fumiquinone B (**205**) displayed antifungal activity against *Diaporthe phaseolorum* spores at a minimum inhibitory concentration of 312 µg/mL, whereas the positive control difenoconazole (commercial fungicide) exhibited MIC at 2.4 µg /mL [113].

### 7.10. Sulphonamide Derivatives

**Figure 15:**
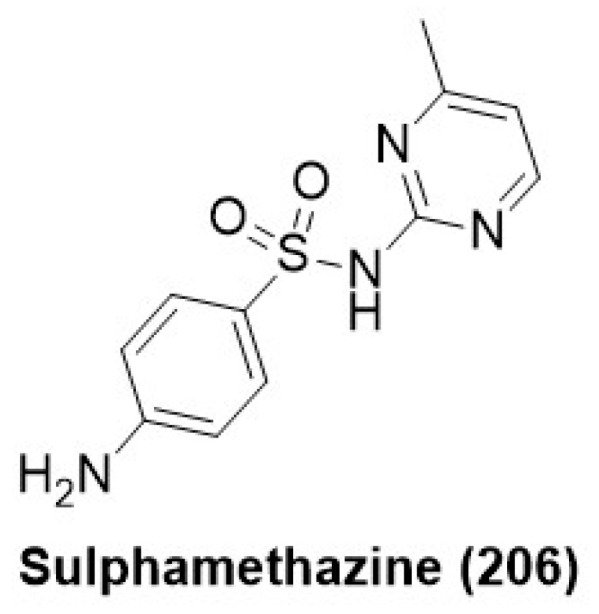
Antifungal Sulphonamide derivative isolated from endophytic Acremonium species (**206**).

Endophytic *Acremonium* species from *Mentha piperita* produced sulphamethazine (**206**) (sulfonamide derivative) (Figure 15). Compound (**206**) displayed good antifungal activity against *Fusarium oxysporum* and *Sclerotinia sclerotiorum*, having growth inhibition of 32.5 and 39.08% at 200 ppm, respectively [114].

## 8. Volatile Organic Compounds (VOCs) as Antifungals

In addition to being prolific producers of diverse secondary metabolites, endophytic fungi also synthesize volatile organic compounds (VOCs). These VOCs play pivotal roles in ecological interactions, including serving as defense mechanisms against pathogens. Consequently, VOCs from endophytic fungi have attracted considerable academic interest due to their potential as natural antifungal agents [115,116,117].

Volatile organic compounds (VOCs) are small, readily evaporative organic molecules that display a range of biological functions, including antimicrobial activity. In the context of endophytic fungi, these VOCs serve dual ecological roles; they aid in the protection of the host plant against pathogens and contribute to the fitness of the fungus within the plant environment [118].

Extensive research has demonstrated the antifungal capabilities of volatile organic compounds produced by endophytic fungi. The volatile organic compounds produced by endophytic fungi can suppress the growth and maturation of phytopathogenic fungi, thus safeguarding the host plant from fungal diseases. The antifungal activity of these VOCs involves diverse mechanisms, potentially including direct toxicity, disruption of fungal cell membranes, or interference with fungal signaling processes [119,120].

**Figure 16:**
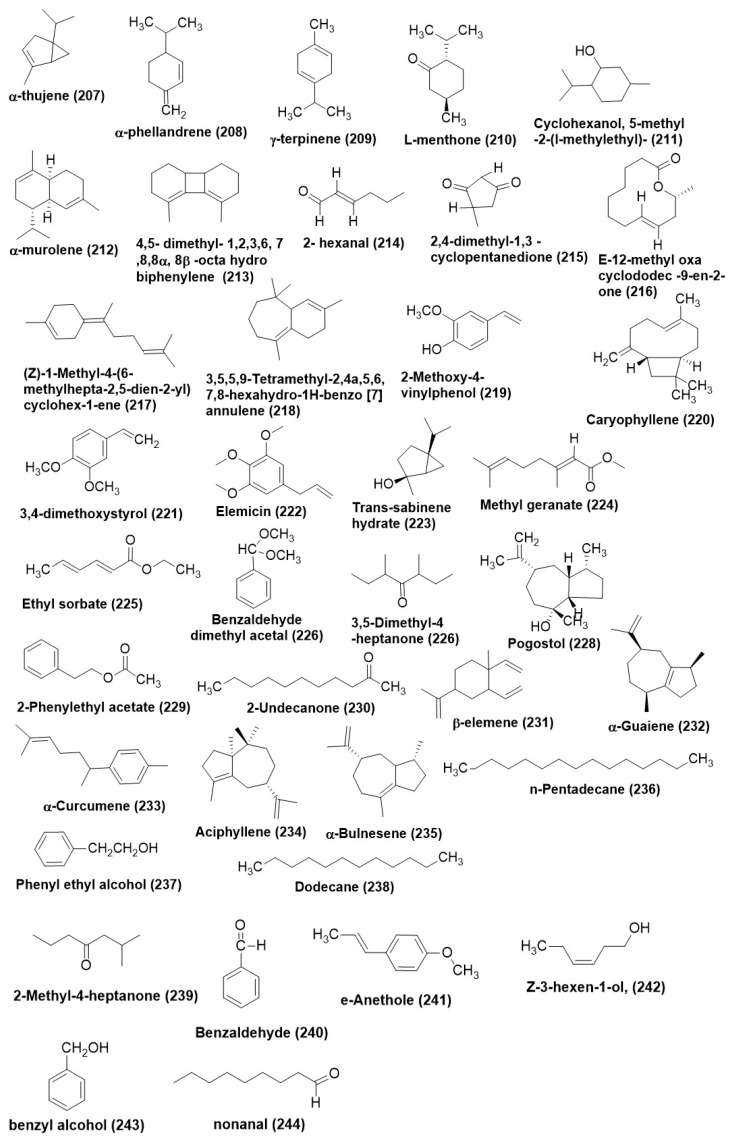
Volatile organic compounds (VOCs) emanated from endophytic fungi possessing antifungal properties (**207**–**244**).

Widespread antifungal activity has been reported from the volatile organic compounds (VOCs) produced by four *Diaporthe* spp. isolated from the medicinal plant *Catharanthus roseus*. The major component of volatile organic compounds (VOCs) comprised of terpenoids apart from other aromatic compounds such as α-thujene (**207**), β-phellandrene (**208**), γ-terpinene (**209**), L-menthone (**210**), Cyclohexanol, 5-methyl-2-(l-methyl ethyl)- (**211**), α-murolene (**212**) (Figure 16). Headspace analysis of *Diaporthe* strains grown on potato dextrose agar revealed that all the strains exhibited a characteristic volatile signature comprising of β-phellandrene (**208**), 4,5-dimethyl 1,2,3,6,7,8,8a,8β-octahydrobiphenylene (**213**) and α-murolene (**212**) (Figure 16) [121].

2-hexanal (**214**) and 2,4-dimethyl-1,3-cyclopentanedione (**215**) (Figure 16) were found in the volatile emissions of the endophytic *Nodulisporium* species PDL-005 isolated from the leaves of medicinal plant *Peperomia dindygulensis*, strongly inhibited *Penicillium digitatum*, apart from *Colletotrichum musa*, *Monilinia fruticola*, *Rhizopus stolonifer* and *Sclerotium rolfsii* [122]. *Cryptosporiopsis ericae* Cc-HG-7 was found to be a potent isolate during a screening program of endophytic fungi from a traditional Chinese medicinal plant, *Coptis chinensis* Franch. for the management of *S. sclerotiorum* and *S. turcica.* Further volatile analysis revealed the presence of four antifungal compounds viz., E-12-methyloxacyclododec-9-en-2-one (**216**), (Z)-1-methyl-4-(6-methylhepta-2,5-dien-2-yl) cyclohex-1-ene (**217**) and 3,5,5,9-tetramethyl-2,4a,5,6,7,8-hexahydro-1H-benzo(7)annulene (**218**) (Figure 16) [123].

Another endophytic fungus, *Sarocladium brachiariae* HND5, was isolated from *Brachiariae brizantha* and was found to produce volatile antifungals, which inhibited the growth of *Fusarium oxysporum* f. sp. *cubense*, an economically important *Fusarium* species. The antifungal VOCs identified through GC-MS were 2-methoxy-4-vinylphenol (**219**), caryophyllene (**220**), and 3,4-dimethoxystyrol (**221**) (Figure 16). Further in vitro studies on the three antifungal VOCs revealed that 2-methoxy-4-vinylphenol (**219**) and 3,4-dimethoxystyrol (**221**) exhibited a more potent antifungal activity as compared to caryophyllene (**220**) [124].

Elemicin (**222**) (Figure 16) is a VOC released by *Daldinia eschscholtzii* MFLUCC 19-0493, an endophytic fungus associated with the plant *Barleria prionitis* and exhibited potential antifungal activity against *Colletotrichum acutatum*. The other antifungal VOCs produced by this endophytic fungus comprised of trans-sabinene hydrate (**223**), methyl geranate (**224**), ethyl sorbate (**225**), benzaldehyde dimethyl acetal (**226**) and 3,5-dimethyl-4-heptanone (**227**) (Figure 16) [125].

The *Muscodor* genus has been a source of a variety of antifungal compounds that have been exploited commercially in both pharmaceutical and agrochemical settings [126]. To date, 22 legitimate species have been described on the basis of their volatile compositions and phylogenetic analysis of the ITS1-5.8S-ITS2 sequences. *Muscodor brasiliensis* sp. nov. LGMF1256 isolated from the Brazilian medicinal plant *Schinus terebinthifolius* prominently produced Pogostol (**228**), 2-phenylethyl acetate (**229**), 2-undecanone (**230**), β-elemene (**231**), α-guaiene (**232**), α-curcumene (**233**), Aciphyllene (**234**), α-bulnesene (**235**) (Figure 16) and n-pentadecane (**236**) (Figure 16). The VOC mixture of the *M. brasiliensis* sp. nov. LGMF 1256 drastically inhibited *Penicillium digitatum*, which is responsible for green mold symptoms in oranges [127].

*Trichoderma* species viz. *T. afroharzianum*, strain MFLUCC19-0090, and *T. afroharzianum* strain MFLUCC19-0091 associated with the medicinal plant *Schefflera leucantha* were found to produce VOCs with prominent activity against *Fusarium oxysporum* and *F. proliferatum* which are also recognized as the major pathogens responsible for post-harvest decay in chilies (fruit). In vitro results exhibited that the volatility emanated from each strain of *Trichoderma afroharzianum* inhibited the pathogen growth. On further GC-MS analysis of the volatile spectrum of both *Trichoderma* strains, prominent VOCs identified were Phenyl ethyl alcohol (**237**)**,** dodecane (**238**), and 2-methyl-4-heptanone (**239**) (Figure 16) in the volatile profile of *T. afroharzianum* strain MFLUCC19-0090 while, phenyl ethyl alcohol (**237**)**,** benzaldehyde (**240**), and e-anethole (**241**) (Figure 16), were detected in high amounts in the volatile profile of *T. afroharzianum* strain MFLUCC19-0091. Phenyl ethyl alcohol (**237**) was identified as the potential antifungal agent inhibiting the growth of *Fusarium oxysporum* as well as *Fusarium proliferatum* [128]. VOC emanated from endophytic *Aureobasidium pullulans* isolated from olive trees is found to produce Z-3- hexen-1-ol (**242**), benzyl alcohol (**243**), and nonanal (**244**) (Figure 16) having strong activity against anthracnose causing fungi, i.e., *Colletotrichum* and *Gloeosporium* species [129].

## 9. Methods Used for Activation of Silent Biosynthetic Genes

Fungal endophytes represent an excellent source of medically important bioactive compounds, demonstrating a wide range of antimicrobial activity against infectious and pathogenic microbes. These secondary metabolites also have different attributes such as immunosuppressant, cholesterol-lowering agent, anti-cancer, anti-inflammatory, anti-diabetic, etc. [130]. Naturally, such compounds are secreted by the microbes for survival or to inhibit the growth of invading enemies [131]. These bioactive compounds are not produced in significant quantity in the axenic culture under laboratory conditions, and the related genes remain silent during the growth of microbial strain in the laboratory. The production of such bioactive compounds thus requires the mimicry of the local habitat to induce or activate the bioactive gene clusters [132]. Many techniques have been developed to activate silent biosynthetic gene clusters, such as epigenetic modification, co-culture technique, and one strain many compounds method (OSMAC) to induce endophytes genome for production of silent secondary metabolites. The details of the above are explained in the anteceding sections.

### 9.1. Epigenetic Modification

Fungal biosynthetic gene clusters contain non-ribosomal peptides, polyketides, ribosomal synthesized post-translationally modified peptides, saccharides, and terpenoids gene clusters that belong to the production of secondary metabolites. This gene cluster structurally differentiated in different domains as non-ribosomal peptides have adenylation, peptidyl carrier protein, and condensation domain, while polyketides have acyl esterase, ketosynthase, and thioesterase domains responsible for secondary metabolites synthesis [133]. The activation of such biosynthetic gene clusters is required for the production of secondary metabolic compounds. Epigenetic modification is one of the powerful tools to activate silent biosynthetic genes in fungi through DNA methylation, RNA interference, and chromatin remodeling through histone modification. In DNA methylation, a methyl group is added to DNA at 5-C of the cytosine ring and converted to 5-methyl cytosine, which inhibits the transcription process [134,135]. Mainly, DNA methylation induces the production of secondary metabolites but rarely reduces the expression of bioactive compounds [136,137]. There are various approaches have been reported to detect the expression of DNA methylation, and the use of inhibitors such as DNA methylase inhibitor 5-azacytidine (5-AC) is one of them. Reduction in the production of aflatoxin and asexual sporulation in *Aspergillus flavus* and *Aspergillus parasiticus* has been reported by the use of 5-azacytidine (5-AC) [138,139]. In fungi, methyltransferase enzymes like DIM-2, Masc2, etc. involved in the DNA methylation process [140]. Similarly, modification in histone through acetylation and deacetylation affects its binding with DNA and expression of the biosynthetic gene cluster. Modification of histone through acetylation changes the chromatin structure and makes it more accessible to promoters of transcription factors, which induce the expression of secondary metabolites and enhance the yield. In contrast, deacetylation of histone condensed the chromatin and inactive the expression of biosynthetic gene clusters [141]. Nicotinamide, Suberoyl bishydroxamic acid (SBHA), and suberoylanilide hydroxamic acid (SAHA) are the most common histone deacetylase inhibitors that inhibit the deacetylation of histone protein [142]. Similarly, regulation of fungal genome by use of various epigenetic elicitors like sodium butyrate, valpronic acid, suberoylanilide hydroxamic acid (SAHA), Trichostatin A (TSA), 5-Azaccytidine, hydralazine hydrochloride and bortezomide in inhibition of histone deacetylase class 1 and 2, DNA methyltransferase and proteosome is low-cost approach to induce silent biosynthetic gene cluster [143].

### 9.2. The Co-Culture Strategy

Another strategy to induce expression in the silent biosynthetic gene cluster is via co-culturing, i.e., growing more than one microorganism in which either a fungal strain is grown with bacteria or with another fungal strain to create a mimic of their natural existence [144]. Various types of interactions, competition, and communication regulate the induction of secondary metabolite production in co-culture techniques. Many reports have been published on the production of novel secondary metabolites through co-culture techniques, including the production of aspergicin by co-culture of two *Aspergillus* species, *Phomopsis* sp. K38 and *Alternaria* sp. E33 on co-culturing led to the production of (3S,7R,8aS)-7-hydroxy-3-(2-methylpropyl)-2,3,6,7,8,8a-hexahydropyrrolo[1,2-α]pyrazine-1,4-dione. The interaction between both co-culture partners is necessary for physical contact or via chemical signals to induce silent gene clusters [145]. In co-cultivation, various low molecular weight molecules have been released and accepted by both partners during communication [146].

### 9.3. OSMAC

One strain many compounds (OSMAC) is an important strategy to express silent biosynthetic gene clusters by modification in culture conditions. Small changes in media composition and growth conditions like pH, oxygen requirement, and temperature significantly affect the metabolic activities of microorganism and their growth [147]. Media is a basic requirement for the growth of heterotrophic microorganisms, and carbon and nitrogen are the most important components of media. Both are important in the synthesis of macromolecules and sources of energy, as well as composing the structural parts of secondary metabolites [148,149]. The C/N ratio in the growth medium also affects the production of secondary metabolites during the fermentation process. Fungus *Cladosporium sphaerospermum* 2005-01-E3 produces five new polyketides in a rice-based medium, while the same strain produced another two polyketides in a soybean flour medium during fermentation [150,151]. The salinity of the medium also affects the induction of silent biosynthetic genes via various halogen groups, which induce the synthetic pathways to restore the osmotic imbalance and activate the silent biosynthetic genes cluster. The use of chemical stressors or elicitors in media also modulates the culture conditions and induces the silent biosynthetic genes to produce secondary metabolites. For example, the addition of heat shock chemicals in media induced the production of jadomycin and validamycin in selected strains during fermentation [152]. Further, various microbial enzymes, soil extracts, antibiotics, metals, and synthetic chemicals have also been used as chemical elicitors to induce silent biosynthetic gene clusters [153].

## 10. Conclusions

Antifungal resistance is a looming danger since the therapeutic interventions to treat systemic and invasive fungal infections are getting limited, thus posing a serious challenge to clinicians. The majority of fungi are opportunistic pathogens and can become pathogenic due to the weak defense status of humans, irrespective of whether the person is an outpatient or an inpatient. Several fungal diseases are hospital-acquired, such as during ventilator support, like the *Candida* species and *Aspergillus fumigatus*. Patients who are immunocompromised or have weak immune status have a high chance of acquiring diseases caused by these opportunistic fungi. The drastic climate change is also one of the presumptive causes of occurrence. This is further aggravated by the presence and use of different antifungals in the environment that render them resistant to the current armamentarium of antifungal drugs**.** Hence, it has become imperative to explore newer avenues in the development of an antifungal armamentarium to tackle the problem of antifungal resistance of these opportunistic pathogens in high concentrations in the environment. A very pertinent example is when a plant pathogenic fungus become opportunistic under the weakened immune status of the human and animal host. Despite the fact that there is a global emphasis on the treatment of superficial and systemic mycoses caused by the genus *Candida* and *Aspergillus* due to their invasive nature and opportunistic approach. However, it has been realized that plant pathogenic fungi may also have a critical impact on human health and safety. Plant pathogenic fungi today are crossing the kingdom border, and several reports exist wherein plant pathogenic fungi have been found to be responsible for a variety of human diseases despite structural and systematic differences between plants and animals. For example, *Alternaria infectoria* is responsible for blossom blight in Guayle. However, the clinical manifestations of this plant pathogen in humans are phaeohyphomycosis and keratitis [154,155]. Tomato head blight is caused by *Fusarium graminiaerum*, which has been found to cause bloodstream infection in humans [156]. *Colletotrichum truncatum*, causing lesions and blight in strawberries and citrus, respectively, has been found to cause ophthalmic (eye) infections in humans [157,158]. Further, several species of *Cladosporium* responsible for plant pathogenesis have been isolated from human clinical samples due to their opportunistic human pathogenic properties [159]. Thus, it is amply clear that plant-pathogenic fungi can cause human and animal infections [160]. Endophytic fungi offer a plethora of non-volatile and volatile bioactive secondary metabolites that serve as an important repository for the development of compounds that are candidates for the development of future antifungal drugs. Hence, through this review, we wish to timely emphasize that all those compounds that are being explored as biofungicides and plant antifungals should be given due credence for their potential to be used in the antifungal drug discovery and development pipeline, given the fact the line of kingdom border between plant and animals has been crossed by these fungi. Further genetic manipulations like epigenetics and OSMAC offer opportunities to activate the silent biosynthetic gene clusters that may diversify the secretome and volatilome of these endophytic fungi to generate novel chemical templates.

## Data Availability

Not applicable.
